# Comparative proteomics of Bt-transgenic and non-transgenic cotton leaves

**DOI:** 10.1186/s12953-015-0071-8

**Published:** 2015-05-02

**Authors:** Limin Wang, Xuchu Wang, Xiang Jin, Ruizong Jia, Qixing Huang, Yanhua Tan, Anping Guo

**Affiliations:** Chinese Academy of Tropical Agricultural Sciences, The Institute of Tropical Biosciences and Biotechnology, Haikou, Hainan 571101 China; Chinese Academy of Agricultural Sciences, The Oilcrops Research Institute, Wuhan, 430062 China

**Keywords:** *Bacillus thuringiensis*, Comparative proteomics, Cotton leaf, Cry1Ac gene, Toxin protein, Transgenic plant

## Abstract

**Background:**

As the rapid growth of the commercialized acreage in genetically modified (GM) crops, the unintended effects of GM crops’ biosafety assessment have been given much attention. To investigate whether transgenic events cause unintended effects, comparative proteomics of cotton leaves between the commercial transgenic Bt + CpTI cotton SGK321 (BT) clone and its non-transgenic parental counterpart SY321 wild type (WT) was performed.

**Results:**

Using enzyme linked immunosorbent assay (ELISA), Cry1Ac toxin protein was detected in the BT leaves, while its content was only 0.31 pg/g. By 2-DE, 58 differentially expressed proteins (DEPs) were detected. Among them 35 were identified by MS. These identified DEPs were mainly involved in carbohydrate transport and metabolism, chaperones related to post-translational modification and energy production. Pathway analysis revealed that most of the DEPs were implicated in carbon fixation and photosynthesis, glyoxylate and dicarboxylate metabolism, and oxidative pentose phosphate pathway. Thirteen identified proteins were involved in protein-protein interaction. The protein interactions were mainly involved in photosynthesis and energy metabolite pathway.

**Conclusions:**

Our study demonstrated that exogenous DNA in a host cotton genome can affect the plant growth and photosynthesis. Although some unintended variations of proteins were found between BT and WT cotton, no toxic proteins or allergens were detected. This study verified genetically modified operation did not sharply alter cotton leaf proteome, and the target proteins were hardly checked by traditional proteomic analysis.

**Electronic supplementary material:**

The online version of this article (doi:10.1186/s12953-015-0071-8) contains supplementary material, which is available to authorized users.

## Background

Since the first genetically modified (GM) crops were commercialized in 1996, the global GM crops have increased more than 100-fold from 1.7 million hectares in 1996 to over 175 million hectares in 2013 [1]. GM crops offer farmers opportunities to improve their products by planting disease resistance, drought resistance or nutrient components which incorporates new genes into crop plants [2,3]. Despite the many benefits of GM crops, the biggest problem is controversial on the safety of food that derived from GM crops. An important issue is whether the existence of unintended effects which are caused by random insertion of exogenous specific genes into plant genomes that may result in disruption, modification or rearrangement of the genome [4,5]. These unintended processes may further result in the formation of new biochemical processes or new proteins (especially new allergens or toxins), which have been an important matter of concerns [6,7]. So, evaluation of whether transgenic events have caused unintended changes is essential to guarantee the food safety and solve the controversial issue on the GM crops.

The concept of substantial equivalence was proposed as a major principle and guiding tool of biological safety assessment according to the Organization for Economic Cooperation and Development [8,9]. Also, more and more approaches involving in targeted and non-targeted genes were applied to assess the safety of GM crops. Traditional methods to detect the safety of GM crops mainly focused on the analysis of key nutritional and non-nutritional components, including the enzyme linked immunosorbent assay (ELISA) and PCR detection of some specific genes, which are considered as targeted approaches [6,10]. At present, non-targeted approaches including the profiling techniques (such as genomics, transcriptomics, proteomics, and metabolomics) allow for simultaneously measuring and comparing the entire sets of transcripts, proteins, and metabolites in organisms [9,11-13]. These non-targeted approaches have been considered to provide unbiased results and more complete insights into any unpredicted changes.

Many studies have been conducted using profiling techniques to evaluate GM crops. Among the profiling techniques, proteomics is a direct method of investigation unpredicted alteration [14,15]. It has a broad application prospects in the safety assessment of genetically modified crops [16]. Proteins are not only the key players in gene function and directly involved in metabolism and cellular development, but also have roles as toxin, antinutrients, or allergens, which have great impact on human health [5,17]. Comparative proteomics by 2-DE combined with mass spectrometry (MS) technologies have been widely used to assess the safety of GM crops, such as soybean [18,19], rice [10,20], maize [8,21-23], potato [24,25], tomato [26,27], and wheat [28,29]. These studies mainly focused on detecting the unintended effects and researching the functional characterization of GM crops. However, no comparative proteomics on GM cotton was reported till now.

Transgenic insect-resistant cotton is the fastest one of global commercialization GM crops because of its economic advantages and environmental impacts, increasing income and reducing environmental pollution by reducing usage of pesticides [30,31]. The global cultivated area of GM cotton was reaching 23.9 million hectares in 2013. Previous studies mainly focused on detecting the biochemical compounds differences between transgenic and non-transgenic cotton, including amino acids fatty acids, carbohydrate content [32]. Fourier transform infrared spectroscopy (FTIR) was also used to detect the chemical and conformational changes between transgenic cotton seeds and their non-transgenic counterparts, and found both the indigenous and exogenous proteins structural changes in genetically modified organism (GMO) [33]. However, it didn’t mention that the transgenic cotton might result in some protein changes and the formation of new metabolites or altered levels of existing metabolites.

Leaves are key organs for plant biomass and seed production because of their roles in energy capture and carbon conversion [34]. In the present study, we carried out comparative proteomics between transgenic cotton line with a toxin CrylAc gene from *Bacillus thuringiensis* (BT) and non-transgenic cotton (WT) leaves combined with 2-DE and MS to study the protein changed level for evaluating the unintended effects in the transgenic cotton. The transgenic cotton lines contain the inserted Cry1Ac and CpTI gene. Hypothetically, the only expected difference between BT and WT should be the presence of BT and CpTI proteins. However, none of these proteins were detected by 2-DE and MS. In addition, none of the DEPs was a toxic protein but related to central carbon metabolism, starch synthesis, protein folding and modification.

## Result

### PCR and ELISA detection of target protein

A 119 bp DNA band only detected in BT leaves by PCR using gene specific primers, confirmed the exist of exogenous CrylAc gene in BT cotton (Additional file 1A). Envirologix’s plate kits for Cry1Ac were used to study expression of Cry1Ac gene in transgenic and the non-transgenic cotton leaves. Cry1Ac expressed protein was not detected in non-transgenic cotton, but was detected at expressed level of 0.31 pg/g in the transgenic cotton leaves (Additional file 1B). The result suggested that Bt toxin protein was really existed in transgenic cotton, but the protein abundance was extremely low. RT-PCR revealed BT line had one detectable DNA fragment with a size of 282 bp. The DNA fragments were undetected in their nontransgenic controls (Additional file 1C).

**Figure 1:**
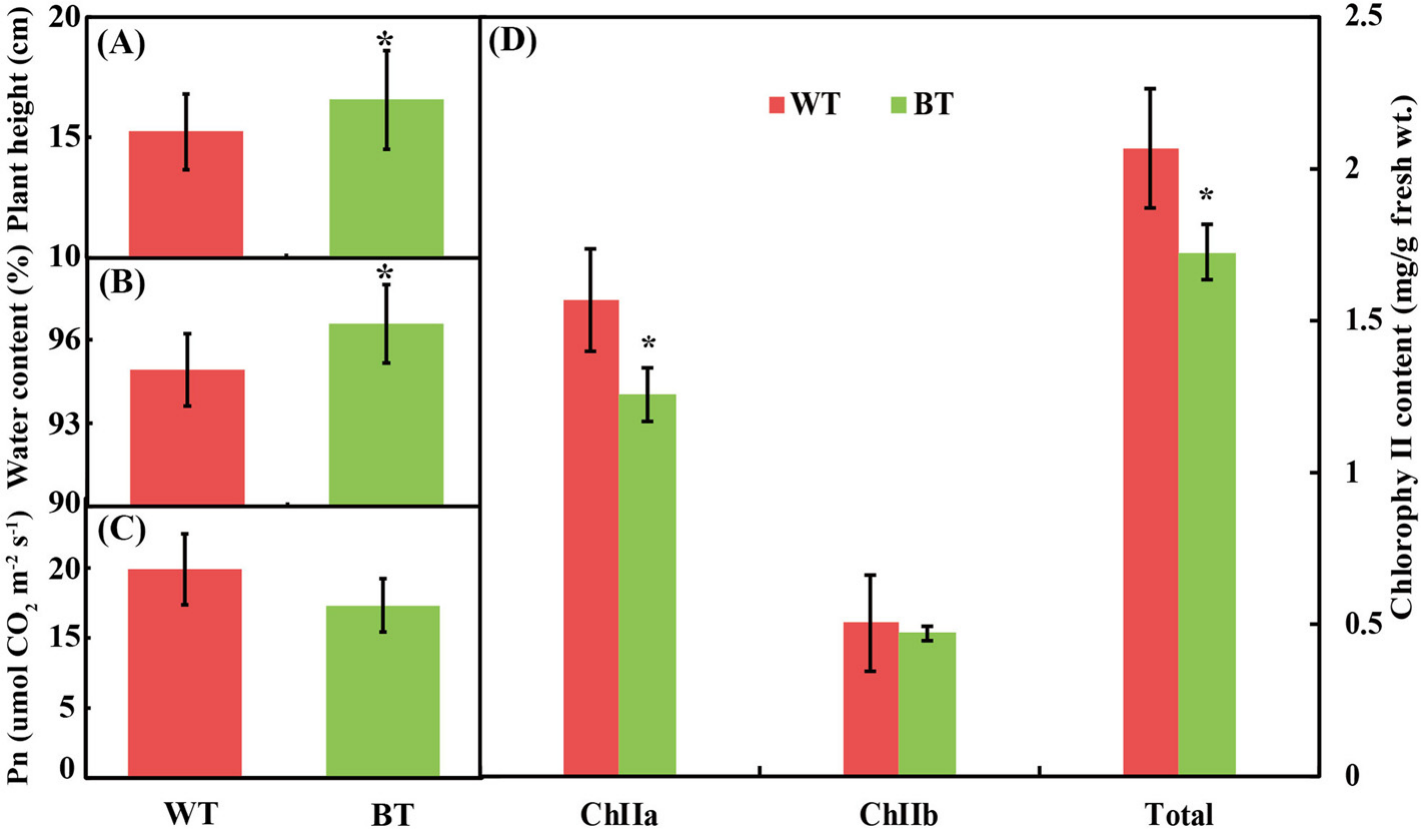
Growth patterns and physiological changes of the Bt-trangenic and non-transgenic cottons. The plant height **(A)**, leaf water content **(B)**, plant net photosynthetic rate **(C)**, and chlorophyll content **(D)** were highlighted. Statistically significant differences relative to the control plants were calculated by independent Student *T*-test. *indicated p < 0.05.

Physiological parameters were compared between WT and BT lines (Figure 1). In BT lines, the plant heights (Figure 1A) and water content (Figure 1B) were significantly increased. In contrast, the net photosynthetic rate (Figure 1C) and chlorophyll content (Figure 1D) decreased in BT lines. The result suggested that the inserted Cry1Ac and CpTI gene directly or indirectly effect the plant growth and photosynthesis.

### Analysis of protein profiles of non-Transgenic and Bt-Transgenic cotton leaves

**Figure 2:**
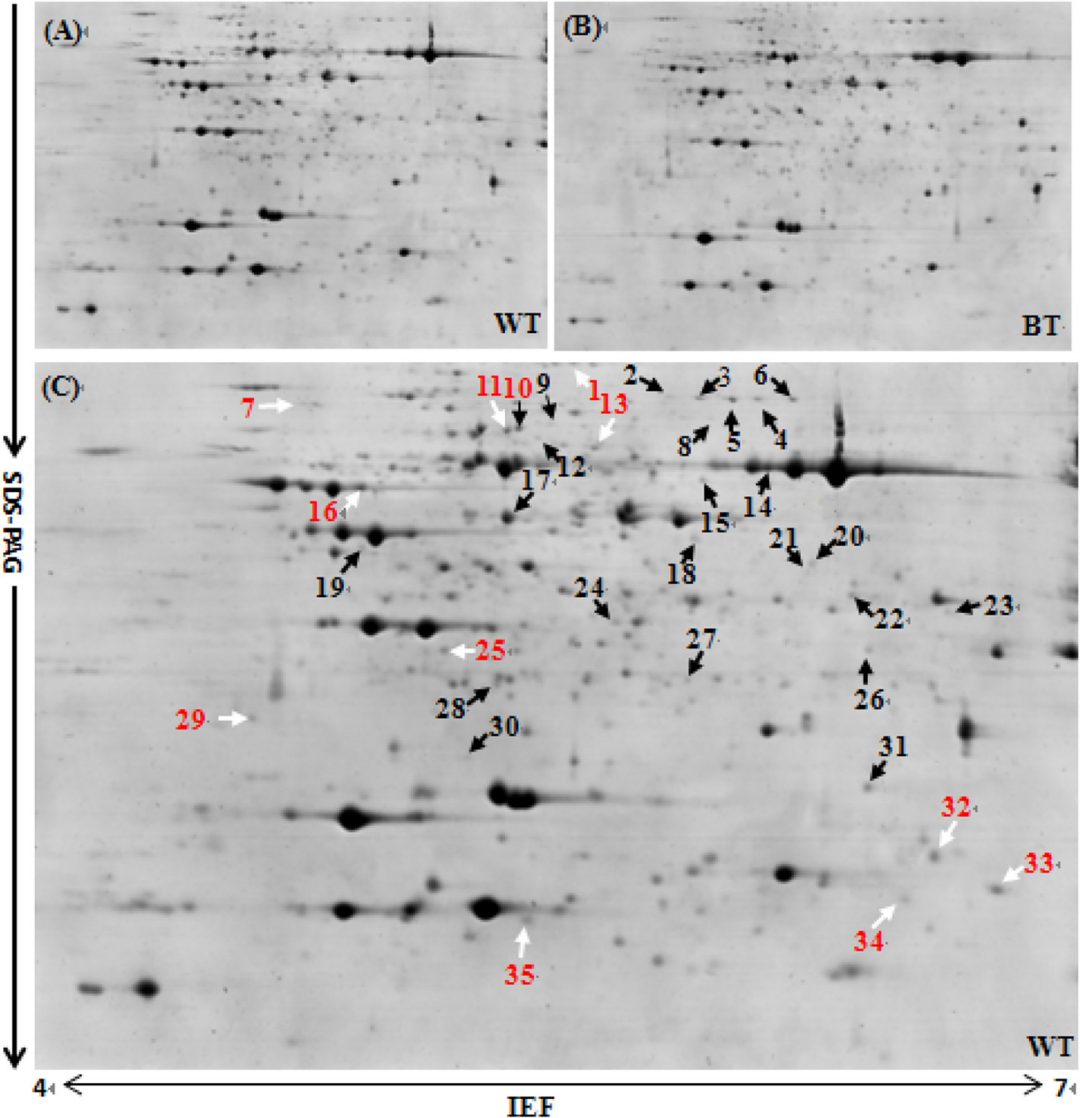
Typical 2-DE profile of leaf proteins from the transgenic cotton line and its control. 2-DE protein profiles of the WT **(A)** and BT **(B)** were presented, and the identified DEPs were marked with the number on the 2-DE gels **(C)**. Arrows indicated the 35 positively identified protein spots by MALDI TOF/TOF MS. Their identities were listed in Table 1 and Additional file 2.

2-DE and image analysis of the protein profiles were carried out to detect the DEPs between the WT (Figure 2A) and BT (Figure 2B) lines. Total proteins of 2-DE reference maps were obtained using IPG strips with pH 4–7 and 12% SDS-PAGE (Figure 2A-C). Protein spots were detected and quantified using Image Master 2D Platinum Software (Version 5.0, GE Healthcare). Our results showed that more than 600 protein spots were detected in each 2-DE image with good reproducibility, respectively. Only the DEPs with abundance change more than 1.5 fold (confidence above 95%, p < 0.05) were selected for MS analysis. Compared to the WT line, a total of 58 DEPs (Figure 2C) were selected, including 34 up-regulated and 24 down-regulated protein spots (Table 1).

**Table 1:** **Proteins identified by MALDI TOF/TOF MS from transgenic cotton leaves**

**Spot no** ^**a**^.	**GI no.** ^**b**^	**Function category protein name**	**Plant species**	**Exper.** ^**c**^ **pI/Mr**	**Thero.** ^**d**^ **pI/Mr**	**M P** ^**e**^	**SC%** ^**f**^	**M. S.** ^**g**^	**Relative change (WT/BT)** ^**h**^
**Posttranslational modification, protein turnover, chaperones (O)**									
1	255556934	ATPase	*R. communis*	5.49/90	5.13/90.1	11	21	324	
7	147819511	Hypothetical protein	*V. vinifera*	4.86/72	5.20/61.4	5	10	256	
9	225433375	Chaperonin	*V. vinifera*	5.40/63	5.85/61.7	6	14	204	
16	12620883	Rubisco activase	*G. hirsutum*	4.99/49	5.06/48.6	13	41	822	
27	14594915	Proteasome subunit	*N. tabacum*	5.77/27	6.12/18.2	3	32	113	
**Carbohydrate transport and metabolism (G)**									
2	110224784	Transketolase	*P. acerifolia*	5.73/76	6.25/26.0	2	11	82	
3	255541252	Transketolase	*R. communis*	5.80/76	6.52/81.6	5	8	277	
4	255541252	Transketolase	*R. communis*	5.95/76	6.52/81.6	5	8	262	
5	255541252	Transketolase	*R. communis*	5.89/76	6.52/81.6	6	10	404	
6	255541252	Transketolase	*R. communis*	6.05/76	6.52/81.6	6	11	337	
8	3560664	Rubisco	*C. ensifolium*	5.73/66	6.4/49.7	5	13	211	
14	11230404	Rubisco, large subunit	*C. Pettersson*	5.96/53	5.96/52.9	12	40	998	
15	33415263	Enolase	*G. hirsutum*	5.69/49	6.16/47.9	11	36	502	
22	329317332	Rubisco, large subunit	*G. barbadense*	6.20/35	6.00/53.7	5	16	326	
24	449442663	Phosphoglycolate phosphatase	*C. sativus*	5.58/32	6.47/41.7	8	26	442	
26	1881499	Rubisco, large subunit	*P. pendula*	6.24/30	6.61/52.6	9	22	366	
32	548699	Rubisco, large chain	(−)	6.40/19	6.12/52.6	4	9	104	
**Energy production and conversion (C)**									
10	40850676	Betaine-aldehyde dehydrogenase	*G. hirsutum*	5.43/63	5.6/55.4	5	12	124	
11	91208909	ATP synthase, beta subunit	*G. hirsutum*	5.33/59	5.22/53.6	13	42	939	
20	225451308	Auxin-induced protein	*V. vinifera*	6.09/39	5.96/37.9	1	3	55	
33	315364830	Ion-sulfur protein	*C. lanatus*	6.54/19	8.45/24.6	3	18	231	
**Cytoskeleton (Z)**									
17	281485191	actin	*P. americana*	5.34/44	5.31/41.9	12	46	984	
**Amino acid transport and metabolism (E)**									
18	211906462	Glutamine synthase	*G. hirsutum*	5.80/43	5.77/39.4	5	16	160	
**Coenzyme transport and metabolism (H)**									
19	449433772	Magnesium-chelatase subunit	*C. sativus*	4.98/40	5.72/46.0	5	15	150	
21	255558669	Porphobilinogen deaminase	*R. communis*	6.07/38	6.55/40.3	3	10	50	
**Inorganic ion transport and metabolism (P)**									
23	225431122	Ferredoxin--NADP reductase	*V. vinifera*	6.46/34	8.91/40.8	7	22	270	
**Cell envelope biogenesis, outer membrane (M)**									
25	292668595	Sanguinarine reductase	*E. californica*	5.18/29	4.97/29.6	2	6	104	
**Nucleotide transport and metabolism (F)**									
34	225457446	Nucleoside diphosphate kinase,	*V. vinifera*	6.41/18	9.28/26.0	3	13	147	
**No related to COG (NO)**									
12	255558986	Hypothetical protein	*R. communis*	5.39/58	8.23/54.9	2	2	69	
13	255558986	Hypothetical protein	*R. communis*	5.55/59	8.23/54.9	2	2	53	
28	226358407	Chlorophyll binding protein	*G. hirsutum*	5.30/27	5.53/25.6	3	20	166	
29	118489712	Unknown	*P. trichocarpa*	4.73/26	4.77/24.4	3	9	156	
30	147767601	Hypothetical protein	*V. vinifera*	5.23/42	8.46/25.5	2	11	59	
31	302595736	Oxygen-evolving enhancer protein	(−)	6.26/22	8.67/28.2	3	9	215	
35	211906510	Major latex-like protein	*G. hirsutum*	5.38/17	5.46/17.2	3	27	116	

^a^Assigned spot number as indicated in Figure 2.

^b^Database GI numbers according to NCBInr.

^c,d^ The experimental (c) and theoretical (d) mass (kDa) and pI of the identified proteins.

^e^Number of the matched peptides (MP).

^f^The amino acid sequence coverage (SC) for the identified proteins.

^g^The Mascot searched score (M. S.) against the database NCBInr.

^h^The relatively changed ratios of protein amount on different 2-D gels.

### Protein identification by MALDI TOF/TOF MS

Among the 58 DEPs, 35 (60.3%) proteins were positively identified *via* MALDI TOF/TOF MS (Figure 2), with 23 up-regulated protein spots and 12 down-regulated ones compared to WT. Among these identified proteins, 30 protein species were assigned to potential functions, and the other 5 protein species were identified as hypothetical proteins or unknown proteins (Table 1; Additional files 2 and 3).

**Figure 3:**
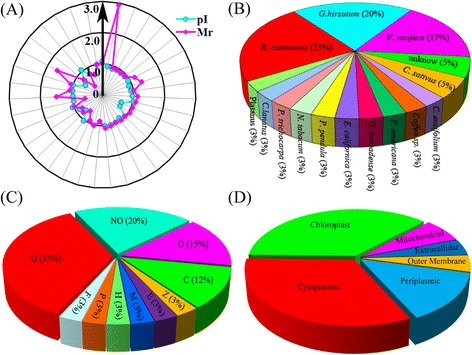
Classification and functional analysis of the identified 35 DEPs. To evaluate the quality of identified proteins, the theoretical and experimental ratios of molecular mass (M*r*) and isoelectric point (*p*I) were determined and presented in radial chart as radial and annular radar axis labels respectively **(A)**. Then, the distributions of the identified proteins in different plant species were also presented **(B)**. Each protein was functionally classified by COG **(C)**. The proportion of each functional category was the sum of the proportion of all identities. The subcellular locations of the identified 35 proteins were presented **(D)**. The abbreviations were: G, carbohydrate transport and metabolism; O, Posttranslational modification, protein turnover, chaperones; C, energy production and conversion; D, Cell division and chromosome partitioning; E, Amino acid transport and metabolism; H, Coenzyme transport and metabolism; P, Inorganic ion transport and metabolism; M, Cell envelope biogenesis, outer membrane; F, Nucleotide transport and metabolism; NO, No related COG.

To evaluate the quality of the proteins identification by MALDI TOF/TOF MS, the theoretical and experimental ratios of molecular weight (M*r*) and isoelectric point (*p*I) were determined, respectively (Table 1). These ratios were presented as radar axis labels (the M*r* ratio for the radial value and the *p*I ratio for the annular value) in radial chart (Figure 3A). When the theoretical and experimental values of the identified proteins are the same, both the radial values and the annular values will be 1.0 and all these identified proteins will be located on the cyclical line 1.0 in radial chart. The closer a spot is to line 1.0, the greater the certainty that the identification made by means of MS/database searching will be the MS identification obtained. More than 80% of the identified protein spots were closely located on the cyclical line 1.0, indicating the high quality of the MS data (Figure 3A).

### Protein function analysis

The identified proteins were obtained from 15 plant species (Figure 3B). The sequence homologies of these identified proteins to those of proteins from other plant species were also determined. Among the identified proteins, 22% showed strong sequence homology to *Ricinus* proteins, followed by 19% of *Gossypium* proteins, and 16% of *Vitis* proteins.

The 35 identified proteins were classified into 10 groups based on their main cellular functions as defined by the COG functional catalogue (Table 1; Figure 3C), including: 35% proteins in carbohydrate transport and metabolism, 15% proteins in chaperones related to post-translational modification, 12% proteins in energy production and conversion, 3% proteins in cell division and chromosome partitioning, 3% proteins in amino acid transport and metabolism, 3% proteins in coenzyme transport and metabolism, 3% proteins in inorganic ion transport and metabolism, 3% proteins in cell envelope biogenesis, outer membrane, 3% proteins in nucleotide transport and metabolism, 20% proteins with no-related or could not be classified by COG classification (Table 1; Figure 3C).

The subcellular locations of the identified 35 proteins were also predicted. Among them, the largest portion including 16 proteins were located in chloroplast. Followed by the 14 proteins which were in cytoplasmic. Then, several proteins were located on the periplasmic, mitochondrial, outermembrane or extracellular (Figure 3D; Additional file 2). These results suggested large number of DEPs related to carbohydrate transport and metabolism mainly located on chloroplast and cytoplasm.

### Pathway analysis of all identified proteins using GO and KEGG

**Figure 4:**
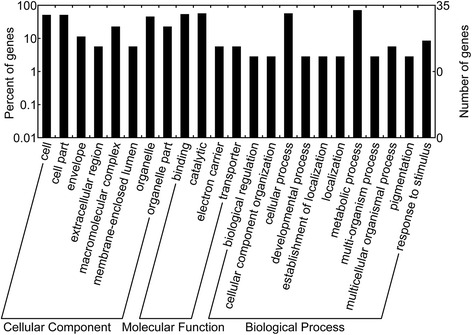
GO classification of the identified DEPs. To reveal the functions of the identified 35 DEPs between WT and BT, GO analysis was performed using WEGO software. The 28 proteins among the identified 35 DEPs were available and then classified into 3 main categories including cellular component, biological process, and molecular function with 23 subgroups. The number of genes denotes that of proteins with GO annotations.

To reveal the functions of DEPs between WT and BT, GO analysis was performed using WEGO software to confirm the cellular component, biological process and molecular function (Figure 4; Additional file 2). Twenty-eight out of the 35 identified proteins were classified into 3 large groups containing 23 subgroups based on their functional annotation. At GO-cellular level, the largest part including 18 proteins were in the cell (GO: 0005623), another 18 proteins occur in the cell part (GO: 0043226), and 16 proteins occur in the organelle (GO: 0043226), with the remainder occurring in the extracellular region (GO: 0005576), membrane-enclosed lumen (GO: 0031974), envelope (GO: 0031975), macromolecular complex (GO: 0032991), and organelle part (GO: 0044422) (Figure 4; Additional file 2). For the molecular function ontology, 4 subcategories were assigned. The largest portion was catalytic activity (GO: 0003824) including 20 proteins, followed 19 proteins with binding function (GO: 0005488). Then, several proteins had transporter activity (GO: 0005215) and electron carrier activity (GO: 0009055) (Figure 4; Additional file 2). In the biological process category, 11 subgroups were over-expressed. The largest part including 25 proteins was related to metabolic process (GO: 0008152), followed by cellular process (GO: 0009987) involving in 20 proteins, with the other important biological processes including cellular component organization (GO: 0016043), multicellular organismal process (GO: 0032501), developmental process (GO: 0032502), pigmentation (GO: 0043473), response to stimulus (GO: 0050896), localization (GO: 0051234), multi-organism process (GO: 0051704) and biological regulation (GO: 0065007) (Figure 4; Additional file 2).

**Figure 5:**
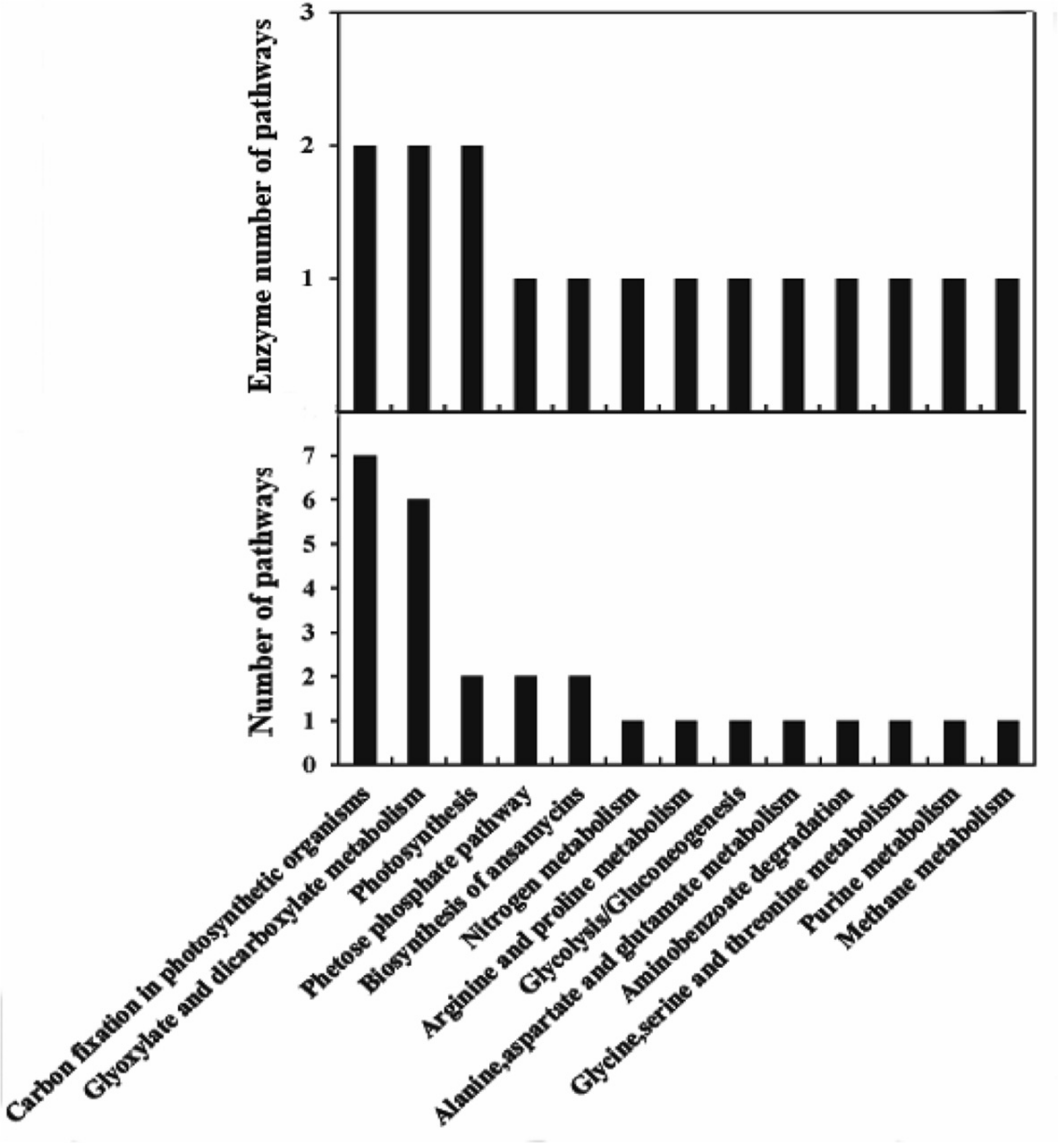
KEGG pathway analysis of the identified 35 DEPs. To determine the molecular interaction and reaction networks of the identified proteins, KEGG pathway analysis was performed. The related pathways were classified into 13 main categories. The number of sequences and enzymes corresponding to each pathway were illustrated.

KEGG pathway analysis was performed using Blast2GO program to determine their molecular interaction and reaction networks and which pathways were significant. The 35 identified proteins were involved in 13 kinds of KEGG pathways (Figure 5; Additional files 4 and 5), including carbon fixation in photosynthetic organisms, glyoxylate and dicarboxylate metabolism, purine metabolism, pentose phosphate pathway, nitrogen metabolism, photosynthesis, oxidative phosphorylation, amino acid metabolism, etc. The most important pathway is carbon fixation in photosynthetic organisms and photosynthesis, which contains 4 identified enzymes named ribulose-bisphosphate carboxylase (EC 4.1.1.39, spots 8, 14, 22, 26 and 32), transketolase (EC 2.2.1.1, spots 2, 3, 4, 5 and 6), ferredoxin--NADP^+^ reductase (EC 1.18.1.2, spot 23), ion-sulfur reductase (EC 1.10.9.1, spot 33). Two enzymes belonging to the porphyrin and chlorophyll metabolism pathway, which are important for photosynthesis in green plants, were also identified. They were hydroxymethylbilane synthase (EC 2.5.1.61, spot 21) and chelatase (EC 6.6.1.1, spot 19). Another important pathway was glyoxylate and dicarboxylate metabolism, for which 3 enzymes were identified from 7 differentially sized protein spots. These enzymes were glutamine synthase (EC 6.3.1.2, spot 18), Rubisco carboxylase (EC 4.1.1.39, spot 8, 14, 22, 26, 32), and phosphatase (EC 3.1.3.18, spot 24). They are key enzymes for carbon fixation and photosynthesis. It is noteworthy that most of enzymes involved in carbon fixation and photosynthesis pathways were considerably up-regulated after target gene over-expression.

### Protein-protein interaction analysis

**Figure 6:**
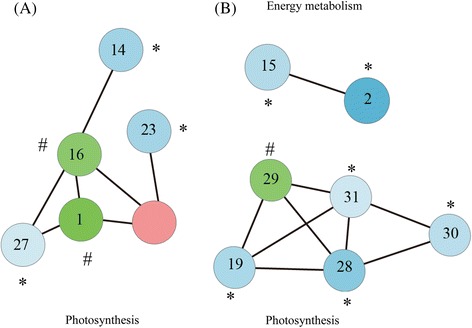
Protein-protein interaction network analysis by STRING. Protein interaction network was generated with STRING and visualized with Cytoscape software. Highly interacting proteins are divided into three clusters which mainly involved in photosynthesis **(A)** and energy metabolism **(B)**. Among them, the 10 up-regulated proteins were marked with * and 3 down-regulated proteins were marked with #.

The DEPs were subjected to STRING database to identify the interaction of these proteins. Protein interaction network was constructed and visualized with Cytoscape software. Among the 35 identified proteins, 13 were involved in protein-protein interaction, and three major clusters of interacting proteins were constructed (Figure 6). The proteins interactions mainly participated in photosynthesis pathway (Figure 6A) and energy metabolism (Figure 6B). Rubisco activase (spot 16) and chlorophyll binding protein (spot 28) are the central core protein of the interacting network, due to their interactions with many other proteins.

### Immunoblot and qRT-PCR analysis

**Figure 7:**
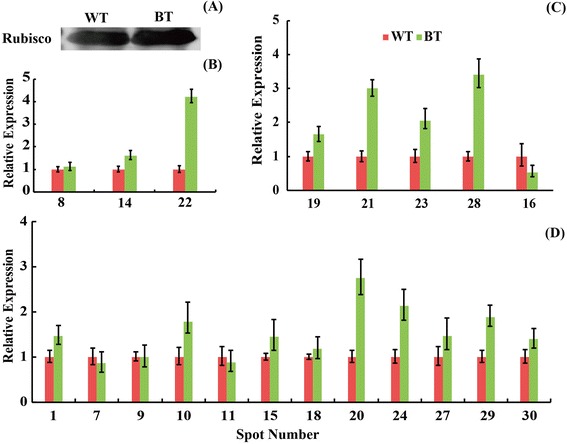
Immunoblot and quantitative RT-PCR analysis of the 20 representative DEPs. The expression profile for Rubisco was detected using 1-D Western blot analysis **(A)**. The identified three different members of Rubisco large subunit genes were up-regulated at transcriptional level in the Bt-transgenic lines **(B)**. The changed patterns of genes for the five DEPs related to photosynthesis were determined **(C)**. The gene expression patterns of the other 12 representative DEPs in both the Bt-transgenic (right) and non-transgenic (left) plants were highlighted **(D)**. The primers used for qRT-PCR were provided in Additional file 6.

Among the DEPs, several proteins with the different molecular weight and *p*I value were identified as Rubisco (spot 8, 14, 22). We used 1-D western blot analysis to determine the expression abundance (Figure 7A). The expression profile showed that a higher level of protein abundance was observed in BT lines.

To explore the changes of DEPs at transcriptional level, 20 representative DEPs were chosen for qRT-PCR to assess their gene expression. The transcriptional expression patterns of these genes were divided into three groups as show in Figure 7B and D. The first group was up-regulated including three genes encoding Rubisco with similar changed pattern both at protein and gene level (Figure 7B). In the second group, DEPs except spot 16 related to photosynthesis were up-regulated with gene encoding Magnesium-chelatase subunit (spot 19), porphobilinogen deaminase (spot 21), Ferredoxin–NADP reductase (spot 23), and chlorophyll binding protein (spot 28) (Figure 7C). The last group displayed the other 12 representative transcripts expression patterns at transcriptional level (Figure 7D). Compared with the expression patterns at transcriptional and translational levels of the 20 coding genes, the transcriptional expression trends of 4 genes named ATPase (spot 1), chaperonin (spot 9), betaine-aldehyde dehydrogenase (spot 10), and a function unknown protein (spot 29) were different with their translational expression. The other 16 genes displayed similar trends at both transcriptional and translational levels.

## Discussion

Since genetically modified crops commercialized, the biosafety assessment of GM crops was concerned by more and more people [35]. To provide more evidence for the biosafety assessment of GM cottons, in this study, we applied proteomics-based approach to investigate the differentially expressed proteins between transgenic cotton leaves and their non-transgenic counterparts. To perform the proteomic analysis, not only the homozygous GM material SGK321, but also the exact non-GM counterpart SY321 was used to minimize the background differences in this study. Also, to ensure that the DEPs mainly come from the transgenic insertion event rather than the genetic background or others, only the protein spots with good reproducibility and which the fold-change in intensity was > 1.5 were further selected to identification *via* MS. Of course, we still cannot exclude the possibility that a few DEPs may come from the genetic background or others, though there was very little possibility. Our results suggested the changes among them were not obviously. The study is consistent with the other GM crops lines finding that no new or toxin proteins were detected in transgenic plants by comparative proteomics [3,8,10,16].

### GM didn’t dramatically alter proteomes of cotton leaves

Some reports referred that random insertion of exogenous genes in plant genomes could lead to disruption of endogenous genes and rearrangement of genome, which could produce new proteins especially new allergens or toxins proteins [10,16]. To evaluate the effected caused Cry1Ac + CpTI genes insertion, 2-DE combining with mass spectrometric techniques was conducted. Approximately 35 DEPs were identified in the transgenic cotton leaves in comparison with their non-transgenic lines. Nevertheless, neither allergens nor BT toxics were detected in transgenic cotton leaves in 2-DE gels. It was possibly due to the low abundance of Cry1Ac protein, which was detected as only 0.31 pg/g in transgenic cotton leaves (Figure 1B), so that it was undetectable in 2-DE gels. Similar result has been noted in other studies. This is expected because proteomics is a useful method for comprehensive analyses but not if the level of a target protein is extremely low. The result implying that GM did not sharply alter the proteome of cotton leaves, and also did not lead to the unintended effects, if it exists, was slight or not easy to detect.

### Carbon fixation in photosynthesis is a major biological process in DEPs

The metabolic variations between the transgenic plant and its non-transgenic line might be due to the position effect of gene insertion [32]. According to the KEGG analysis, the present results revealed that DEPs between WT and BT lines mainly involved in photosynthetic organisms to take part into carbon fixation, photosynthesis, glyoxylate and dicarboxylate, oxidative phosphorylation, pentose phosphate pathway, and so on (Additional files 4 and 5). The largest portion of metabolic-related DEPs whose abundance changed significantly was connected with carbon fixation in photosynthetic organisms and photosynthesis. The unintended variations and effects could have effects on plant growth and developments. Photosynthesis is the process that plant converts light energy into chemical energy including light reaction and carbon reaction (dark reaction). It is not only the basis of biological survival, but also an important to meet energy and food needs. The recent in basic and applied research on photosynthesis more and more focused on the carbon fixation efficiencies improvements, due to the crops yield and energy requirement [36]. Our research revealed that 1 ribulose-bisphosphate carboxylase (Rubisco) (spots 8), 4 Rubisco large subunits (spots 14, 22, 26 and 32) and 5 transketolases (spots 2, 3, 4, 5 and 6) participated in the carbon fixation, with more expression in transgenic cotton line except for spot 32 (Table 1; Additional files 4 and 5). Rubisco has a pivotal role in photosynthetic organisms [37]. This enzyme catalyzes the carboxylation step in the Calvin cycle of carbon fixation, accompanying the process that stores the energy trapped by photosynthesis and also catalyzes the oxygenation step in photorespiration, during which a considerable amount of the stored energy is converted to heat thereby limiting crop yield [38]. In this study, most large subunits of Rubisco showed to be increased at both protein expression abundance and transcriptional expression patterns in the transgenic cotton lines (Table 1; Figure 7A and B), suggesting the efficiency of CO_2_ fixation is increased in transgenic cotton. Additionally, 5 ribulose-bisphosphate carboxylases (spots 8, 14, 22, 26 and 32) also took part in the glyoxylate and dicarboxylate metabolism. In plants, transketolase related to energy metabolism can catalyze reactions in the Calvin cycle of photosynthesis and oxidative pentose phosphate pathway (OPPP). Related researches showed reduction of transketolase expression had a marked inhibited on photosynthesis, secondary metabolism, and plant growth but OPPP activity was not strongly inhibited by decreased transketolase activity [39]. In the present study, expression abundance of 5 transketolase isoforms (spots 2, 3, 4, 5 and 6) was up-regulated, implying the transgenic cotton could enhance photosynthesis ability.

In addition, the other related to photosynthesis and energy metabolism proteins also were identified and showed higher abundance in the transgenic cotton. Chlorophyll A-B binding protein is an important component in the light harvesting complex, and is considered as one of the most abundant proteins in chloroplast of plants [40,41]. Its key function is to collect and transfer light energy to photosynthetic reaction center [42]. In our experiment, the abundance of chlorophyll A-B binding protein increased in transgenic cotton line, but the chlorophyll content and Pn decreased in the transgenic cotton. These results demonstrate that photosynthesis changed in the Bt-transgenic line. The unintended effect could be caused by random insertion of exogenous Cry1Ac and CpTI genes in plant genomes. Enolase is a glycolytic enzyme that is responsible for the ATP-generated conversion of 2-phosphoglycerate to phosphoenolpyruvate [43]. In transgenic cotton leaves, the increased enolase helped to the need of cells for extra energy to deal with insertion of exogenous genes. These data revealed that the DEPs related to carbon fixation in photosynthesis organisms and photosynthesis, glyoxylate and dicarboxylate metabolism pathway, oxidative pentose phosphate pathway and energy metabolism were up-regulated, thus resulting in the higher photosynthesis ability in transgenic cotton line, which need further evidence to confirm. In contrast, the net photosynthesis rate decreased in BT lines as shown in Figure 1C. The results suggested the inserted Cry1Ac and CpTI genes can directly or indirectly affect the plant growth and photosynthesis.

## Conclusions

In conclusion, our comparative proteomic data suggested the GM operation did not sharply alter cotton leaf proteome. Less than 10% of 2-DE detectable protein spots were DEPs, which mainly involving in carbon fixation and photosynthesis, glyoxylate and dicarboxylate metabolism pathway, oxidative pentose phosphate pathway. Our data demonstrated that exogenous DNA into a host cotton genome effected the plant growth and photosynthesis.

## Materials and methods

### Plant materials

The transgenic *Bt* + CpTI cotton SGK321 (BT) and their non-transgenic parental counterparts SY321 (WT) were used as the host plants in all experiments. The SGK321 plant species was bred by introducing the synthetic Cry1Ac gene and modified CpTI (cowpea trypsin inhibitor) gene into the cotton cultivar SY321 by way of the pollen tube pathway technique [44]. Then, SGK321 were self-pollinated to obtain homozygous BT plants. Also, the cotton cultivar SGK321 has been developed into a homozygous cotton species science 1999 and were planted commercialized with a new crop species number 2001ED782014 in china since 2002 [45]. Seeds of transgenic Cry1Ac and CpTI cotton cultivar SGK321 and their non-transgenic parental counterparts SY321 were obtained from Biotechnology Research Center of Chinese Academy of Agriculture Sciences. The seeds were germinated in the plastic pots containing 1:1 (v/v) mixture of vermiculite and nutrient soil moistened with distilled water in a growth chamber maintained at a thermo period of 30/22°C of day/night temperature, under long-day conditions (16 h of light and 8 h of dark) and a relative humidity 65 ± 5%. After germination, seedlings were irrigated weekly with Hoagland’s nutrient solution. One month after germination, the cotton leaves were harvested for physiological and proteomic analyses.

### PCR, ELISA and RT-PCR detection

Genomic DNA from transgenic cotton leaves and their non-transgenic controls were extracted using cetyl trimethyl ammonium bromide (CTAB) method as described [46]. PCR analysis was performed to confirm the presence of the exogenous gene Cry1Ac in the transgenic cotton leaves. PCR reactions were carried out in 25 μl volume containing 12.5 μl 2X Taq PCR Master Mix (Trans Gene), 0.5 μl 10 pm/μl of each primer, 2.5 μl 10 ng/μl of template DNA, and 9 μl sterilized H_2_O. The cry1Ac gene-specific primers used were Cry1Ac F (5’-GTTCC AGCTA CAGCTA CCTCC-3’) and Cry1Ac R (5’-CCACT AAAGT TTCTA ACACC CAC-3’) with expected PCR products size 119 bp. The amplification program was performed as follows: initial denaturation at 94°C for 5 min followed by 40 cycles of 45 s at 94°C for denaturation, 45 s at 56°C for primer annealing, 60 s at 72°C for elongation, final elongation at 72°C for 10 min. PCR amplification products were separated using agarose gel electrophoresis in 1X TAE buffer.

The Bt toxin protein content in cotton leaves was measured by ELISA using the Quantiplate Kit for Cry1Ab/Cry1Ac (Envirologix, Inc., USA), which was precoated with Cry1Ac antibody containing 96 well solid microplates. The ELISA experiment was performed according to the protocols provided by manufacturers. Absorbance was measured at 450 nm using a Varioskan Flash Spectral Scan Multimode Plate Reader (Thermo Fisher Scientific, Waltham, MA). A standard curve was established using Cry1Ac standard protein at concentration ranged from 0.1 to 0.5 pg/ml.

Total RNA was isolated to generate cDNA using Reverse Transcriptase kit reagents (TaKaRa, Tokyo, Japan). RT-PCR was used to detect the CPTI gene. The CPTI gene-specific primers were CPTI F (5’-GATTTGAACCACCTCGGAGG-3’) and CPTI R (5’-CTCATCATCTTCATCCCTGG-3’).

### Determination of plant height, water content, photosynthetic rate and chlorophyll content

The plant height was determined immediately after harvesting. The cotton leaves were collected and immediately weighed (fresh weight (FW)). Dry weight (DW) was determined by oven drying at 60°C for 72 h. The total water content (TWC) was calculated as follows: TWC = [(FW-DW)/FW]*100. The collected cotton leaves were washed, cut in small pieces, and ground in 80% chilled acetone. The supernatant was taken for determination of photosynthetic pigments: chlorophylla (mg/g) = (12.7*A663-2.69*A645) V/W, chlorophyllb (mg/g) = (22.9*A645-4.68*A663) V/W, chlorophyll Total (mg/g) = (8.02*A663 + 20.21*A645) V/W. The net photosynthetic rate (Pn) was measured using a LI-6400 Portable Photosynthesis System (Li-Cor, Lincoln, NE, USA) with chamber setting of 400 ppm. And, photosynthetic photon flux density (PPFD) was set at 1000 umol m^−2^ s^−1^.

### Protein preparation

Total leaf protein was extracted using TCA-acetone precipitation method as described [47]. Approximately 1 g of lyophilized powders was precipitated by 10 ml acetone solution containing 10% (w/v) TCA and 0.07% (w/v) β-mercaptoethanol. The mixture was stored at −20°C for 10 h and centrifuged at 15,000 g at 4°C for 30 min to collect precipitates. The precipitates were resuspended by acetone solution containing 0.07% (w/v) β-mercaptoethanol. The mixture was stored at −20°C for 1 h and centrifuged at 15,000 g at 4°C for 30 min to collect the precipitates. The proteins were collected from precipitates by centrifugation at 15,000 g at 4°C for 30 min, washed with 100% ice-cold methanol twice and 100% ice-cold acetone twice, and then air-dried. Resulting proteins were dissolved in lysis buffer (7 M urea, 2 M thiourea, 2% CHAPS, 13 mM DTT) for 2 hours at room temperature. Protein concentration was determined by the Bradford assay using a UV-160 spectrophotometer (Shimadzu, Kyoto, Japan) and bovine serum albumin as the protein standard [48]. The proteins underwent 2-DE immediately or were stored at −80°C.

### 2-DE and image analyses

2-DE was performed according to the manufacturer’s instruction (2-DE Manual, GE Healthcare). A total of 1,200 μg proteins mixed with lysis buffer (7 M urea, 2 M thiourea, 2% CHAPS, 13 mM DTT) were loaded onto an IPG (immobilized pH gradient) strips with linear pH gradient 4–7 and 24 cm length (GE Healthcare, Uppsala, Sweden). The strips were hydrated for 18 h at room temperature. Then isoelectric focusing was performed on an Ettan IPGphor isoelectric focusing system (GE Healthcare, Uppsala, Sweden) under the following conditions: 250 V for 3 h, 500 V for 2 h, 1000 V for 1 h, a gradient to 8000 V for 4 h, and 8000 V up to 140000 Vhr. Subsequently, these strips were equilibrated with equilibration solution (50 mM Tris–HCl, pH 8.8, 6 M urea, 30% glycerol, 2% SDS, 0.002% bromophenol blue) containing 1% DTT for 15 min, followed with equilibration for another 15 min in alkylation buffer containing 50 mM Tris–HCl, pH 8.8, 6 M urea, 30% glycerol, 2% SDS, 0.002% bromophenol blue, and 4% iodoacetamide. Then, IPG strips were transferred to SDS-PAGE gels for separating proteins with an Ettan Dalt system (GE Healthcare). Program was set up as follows: 4 W/gel for 1 h and then 8 W/gel for 6 h [49]. After electrophoresis, the gels were visualized by GAP staining methods [50]. Image analysis was performed using Image Master 2D Platinum Software (Version 5.0, GE Healthcare). The apparent molecular weight (M*r*) of each visible protein was determined through comparison with protein markers with known M*r* values. Biological variation analysis module was employed to identify spots differentially expressed (more than 1.5 fold) in different salt treated samples with statistically significant differences (confidence above 95%, p < 0.05). Three biological repeats for each sample were examined, and the results were shown in average ± SD (n = 3). Then, spots of interests were manually excised from the GAP stained 2-DE gels.

### In-Gel trypsin digestion

The collected protein spots were washed with MilliQ water three times, for 30 min each until removing impurities on the surface of gels. Then, protein spots were destained three times with destaining solution containing 50 mM NH_4_HCO_3_ and 50% ACN for 30 min each at 37°C, and then incubated in 100 μL of 100% ACN until gel pieces became white and shrunken. They were air dried at room temperature for 1 h. Proteins were digested in-gel with bovine trypsin (Roche, Cat. 11418025001) as described [51]. After digestion, the remaining trypsin buffer were discarded, and then centrifuged at 10,000 g for 30 min to collect peptides extracts. 1 μL of peptides extracts was mixed with 1 μL of α-cyano-4-hydroxycinnamic acid (CHCA) and spotted on the target plate.

### Protein Identification *via* MALDI TOF/TOF MS

Proteins were identified by using AB SCIEX MALDI TOF-TOF 5800 system (AB SCIEX, Foster City, CA, USA) equipped with a neodymium with laser wavelength 349 nm as described [51,52]. The laser can shot at a rate of up to 1000 Hz. CHCA was used as the matrix with TFA for an ionization auxiliary reagent. The spectrum was calibrated using the TOF/TOF calibration mixtures (AB SCIEX). All peptide mass fingerprint spectra were internally calibrated with trypsin autolysis peaks, and all known contaminants were excluded during this process. Peptide mass was used to database search.

### Database searching

The raw MS and MS/MS data were combined to search against the taxonomy of Viridiplantae (Green Plants, including 1,196,615 sequences) in NCBI (NCBInr) database with 23,290,086 sequences using an in-house MASCOT server. The searched parameters were set as followings: one missed cleavage, P < 0.05 significance threshold, 100 ppm mass tolerance for precursor ions, MS/MS ion tolerance of 0.1 Da, carbamidomethylation of cysteine as fixed modification, and oxidation of methionine as variable modification. When individual ions scores were higher than threshold score (scores higher than 45), proteins were considered as confident identifications or extensive homology (p < 0.05). For protein scores confidence intervals above 95%, In-house BLAST search against NCBI (http://www.ncbi.nlm) was performed to confirm the protein identifications. The identified proteins were categorized to specific processes or functions by searching Gene Ontology (http://www.geneontology.org) [52].

### Bioinformatic analysis

The cluster of orthologous groups of proteins (COG) analysis was carried out for the identified proteins. Following subcellular localization was predicted using CELLO V.2.5 (http://cello.life.nctu.edu.tw), which made predictions based on a two-level support vector machine system [53,54]. The sequences of the identified proteins were searched against the UniProt database in order to extract corresponding GO information [55]. Then, GO classification of these proteins was conducted with WEGO web service (http:// wego. genomics. org.cn), by which GO terms assigned to query sequences and catalogued groups were produced based on biological process, molecular functions, and cellular components [56-59]. In addition, identified proteins were further analyzed using the STRING V.9.1 database for protein-protein interactions, to statistically determine the functions and pathways most strongly associated with the protein list [60]. Finally, KEGG (http://www.genome.jp/kegg/pathway) pathway analysis was performed to determine their molecular interaction and reaction networks.

### Western blotting analysis and quantitative Real-time PCR

Western blotting was performed as described [61]. About 10 ug proteins were subjected to SDS-PAGE, transferred to a membrane. The 5% nonfat milk was used for blocking protein. The blocked membranes were incubated with specific antibodies against Rubisco at the dilution of 1:8000 at 37°C for 1.5 h. Antibody-bound proteins were detected using appropriate HRP-conjugated secondary antibodies (Sigma, USA) and clarity western ECL substrate (Bio-Rad, CA, USA). The target proteins were then visualized and quantitated using a LAS- 4000 luminescent image analyzer.

Total RNA was isolated to generate cDNA using Reverse Transcriptase kit reagents (TaKaRa, Tokyo, Japan). The primer pairs used for quantitative Real-time PCR (qRT-PCR) are provided in additional file 6. qRT-PCR was performed in a 20ul volume containing 10 ul 2*GoTaq q PCR Master Mix, 2 ul of cDNA, 0.4 ul of each gene-specific primer, 7 ul of Nuclease-Free Water, and 0.2 ul of 100* CXR (Promega, Madison, WI). Reaction was conducted on an Mx3500P Real-Time PCR Detection System according to the manufacturer’s instructions. All data were analyzed using MxPro software.

## Additional files

Additional file 1:
**PCR, ELISA and RT-PCR analysis of Cry1Ac in different cotton leaves.**
Additional file 2:
**MS identification and bioinformatic analysis of the differentially expressed proteins.**
Additional file 3:
**Supplemental spectra and MALDI TOF/TOF MS identification of the differentially expressed proteins.**
Additional file 4:
**Blast2go analysis results of the identified proteins.**
Additional file 5:
**The main pathways involved in transgenic cotton.**
Additional file 6:
**Primers used in qRT-PCR.**

## Abbreviations

ELISA: Enzyme linked immunosorbent assay
DEPs: Differentially expressed proteins
GM: Genetically modified
FTIR: Fourier transform infrared spectroscopy
GMO: Genetically modified organism
CTAB: Cetyl trimethyl ammonium bromide
