# Supplementary material for: Comparative proteomics of Bt-transgenic and non-transgenic cotton leaves
Source: Proteome Sci. 2015 May 2;13:15. doi: 10.1186/s12953-015-0071-8 (PMC4422549; doi:10.1186/s12953-015-0071-8)

**Additional File 1:**

**PCR, ELISA and RT-PCR analysis of Cry1Ac from cotton leaves**

The PCR product of Cry1Ac gene was presented in agarose gel (A). Cry1Ac toxin protein content in the transgenic and non-transgenic cotton leaves was determined using ELISA approach (B). The CPTI gene was detected using RT-PCR (C).


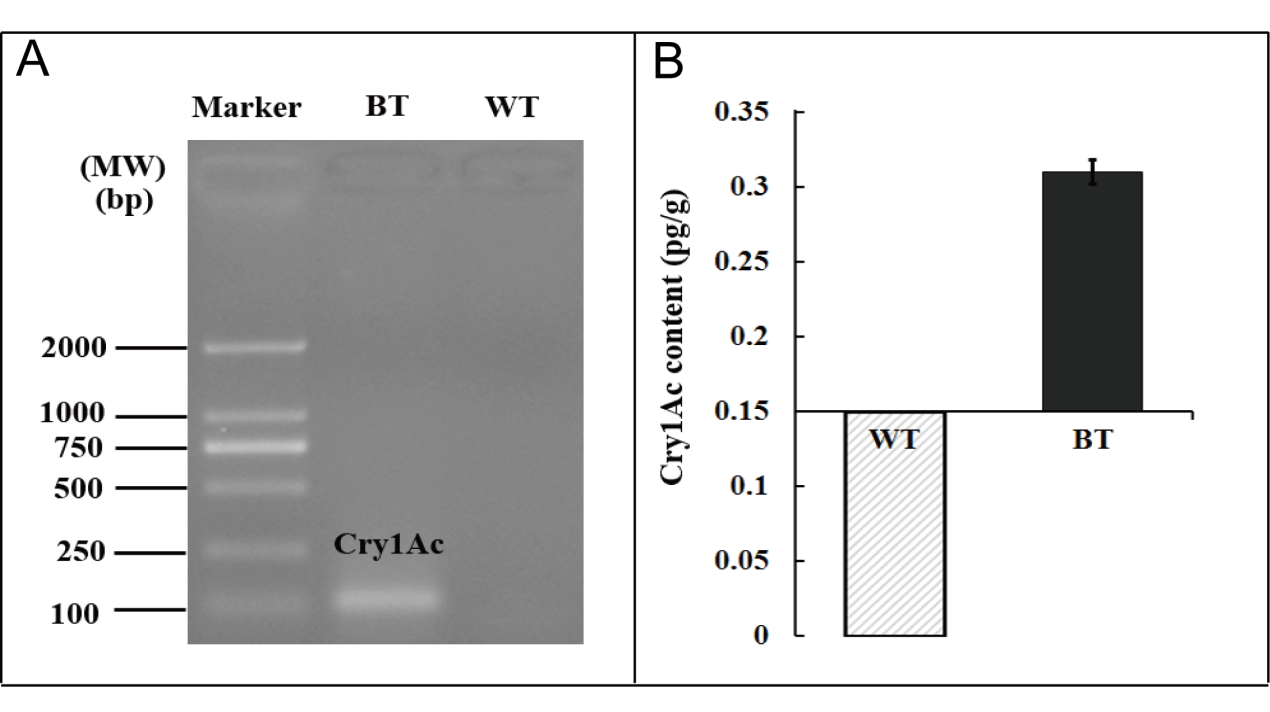


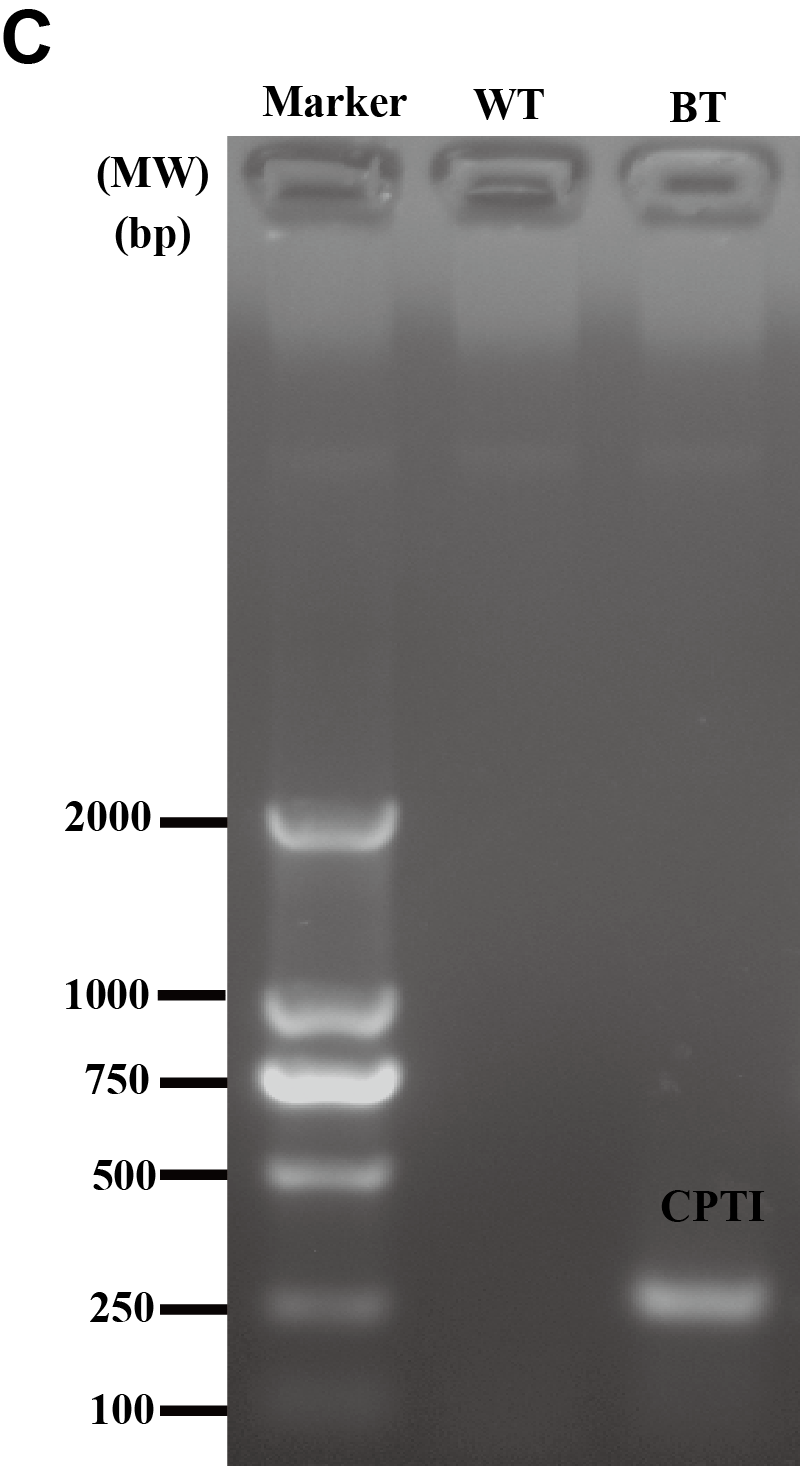

Supplement: Additional file 1: — PCR, ELISA and RT-PCR analysis of Cry1Ac in different cotton leaves. [file 12953_2015_71_MOESM1_ESM.doc]
