# Supplementary material for: Comparative proteomics of Bt-transgenic and non-transgenic cotton leaves
Source: Proteome Sci. 2015 May 2;13:15. doi: 10.1186/s12953-015-0071-8 (PMC4422549; doi:10.1186/s12953-015-0071-8)
Supplement: Additional file 3: — Supplemental spectra and MALDI TOF/TOF MS identification of the differentially expressed proteins. [file 12953_2015_71_MOESM3_ESM.doc]

**Additional File 3:**

**Supplemental spectra and MALDI TOF/TOF MS/MS identification of the differentially expressed proteins.**

**Annotated spectra for Table 1: 35 proteins identified by PFF.**

Spot numbers of the 35 proteins correspond to the proteins that listed in Table 1.

**CID:** collision induced dissociation

**MALDI TOF**:

matrix assisted laser desorption/ionization time of flight

**MS**: mass spectrometry

**PFF**: peptide fragment fingerprinting

Spot No.: **1**

NCBI accession No.: gi | **225440045** Species: *Ricinus communis*

PFF score: [324]

Protein name: Transitional endoplasmic reticulum ATPase

Matched peptides No.: [11] Sequence coverage %: [21]

Calculated Mr: **50196** Calculated *p*I: **5.14**

Probability Based Mow


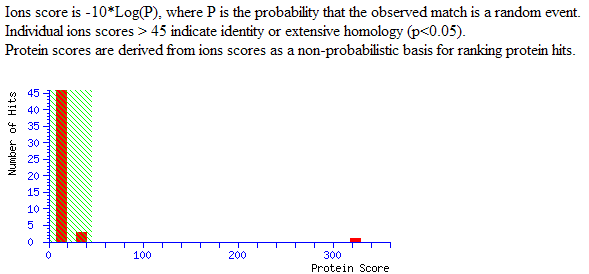


Matched peptide sequences: shown in Bold Red


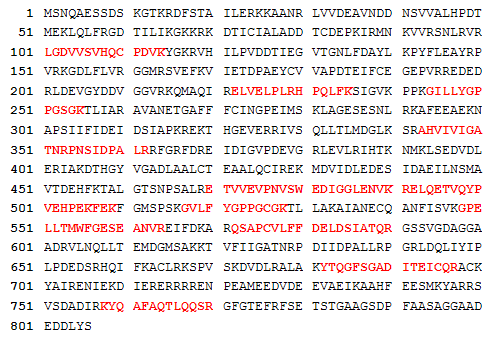


Matched peptides information:


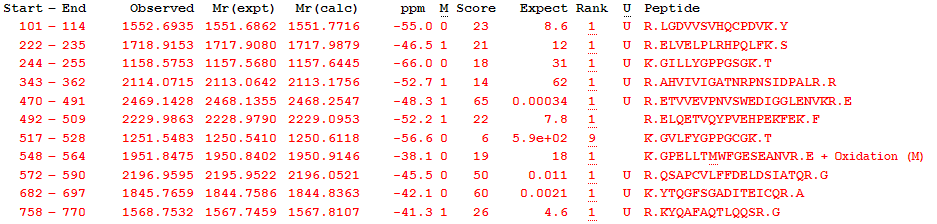


Spot No.: **2**

NCBI accession No.: gi | **110224784** Species: *Platanus x acerifolia*

PFF score: [82]

Protein name: transketolase

Matched peptides No.: [2] Sequence coverage %: [11]

Calculated Mr: **25954** Calculated *p*I: **6.25**

Probability Based Mow


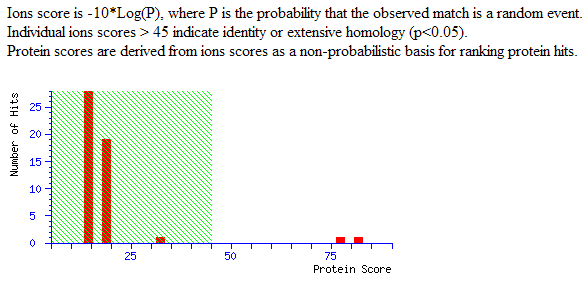


Matched peptide sequences: shown in Bold Red


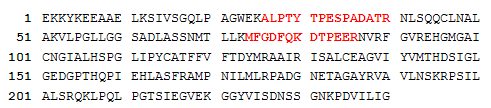


Matched peptides information:


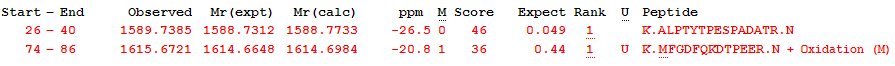


Spot No.: **3**

NCBI accession No.: gi |**255541252** Species: *Ricinus communis*

PFF score: [277]

Protein name: transketolase

Matched peptides No.: [5] Sequence coverage %: [8]

Calculated Mr: **81623** Calculated *p*I: **6.52**

Probability Based Mow


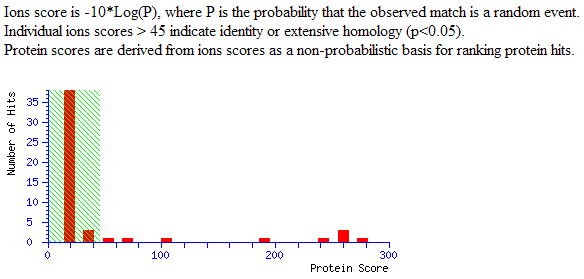


Matched peptide sequences: shown in Bold Red


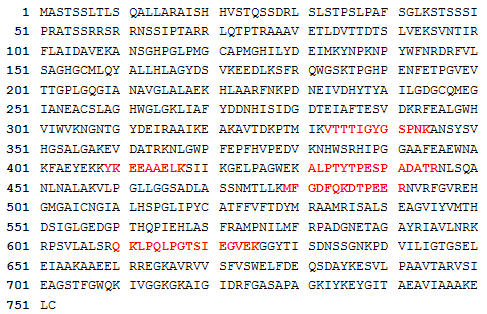


Matched peptides information:


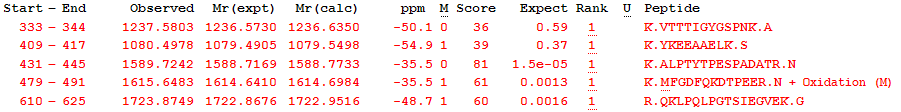


Spot No.: **4**

NCBI accession No.: gi | **255541252** Species: *Ricinus communis*

PFF score: [262]

Protein name: transketolase

Matched peptides No.: [5] Sequence coverage %: [8]

Calculated Mr: **81623** Calculated *p*I: **6.52**

Probability Based Mow


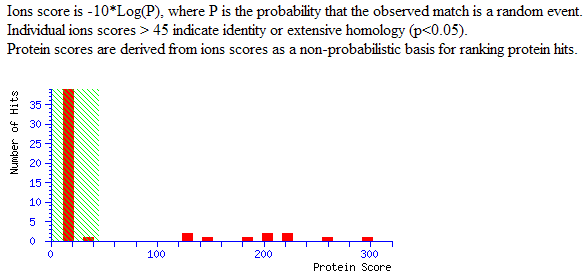


Matched peptide sequences: shown in Bold Red


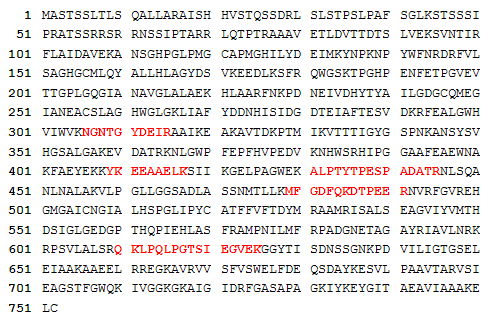


Matched peptides information:


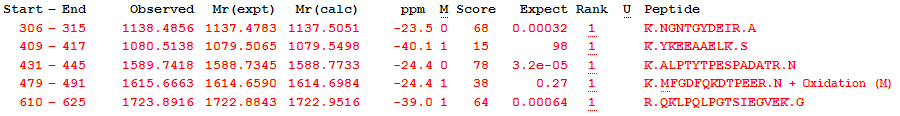


Spot No.: **5**

NCBI accession No.: gi | **255541252** Species: *Ricinus communis*

PFF score: [404]

Protein name: transketolase

Matched peptides No.: [6] Sequence coverage %: [10]

Calculated Mr: **81623** Calculated *p*I: **6.52**

Probability Based Mow


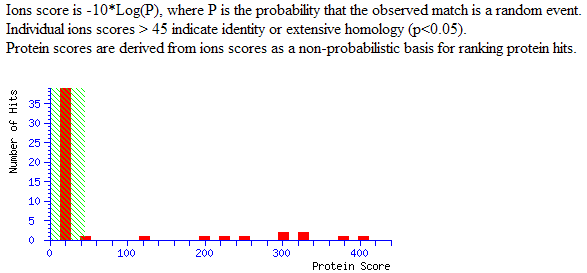


Matched peptide sequences: shown in Bold Red


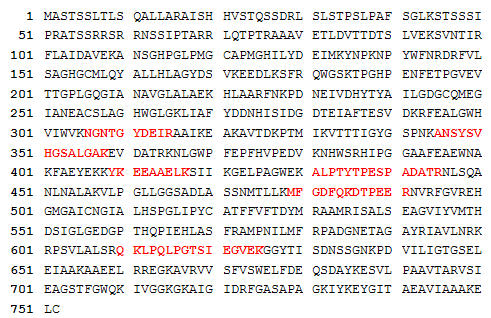


Matched peptides information:


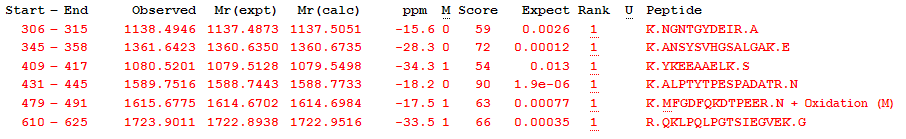


Spot No.: **6**

NCBI accession No.: gi | **255541252** Species: *Ricinus communis*

PFF score: [337]

Protein name: transketolase

Matched peptides No.: [6] Sequence coverage %: [11]

Calculated Mr: **81623** Calculated *p*I: **6.52**

Probability Based Mow


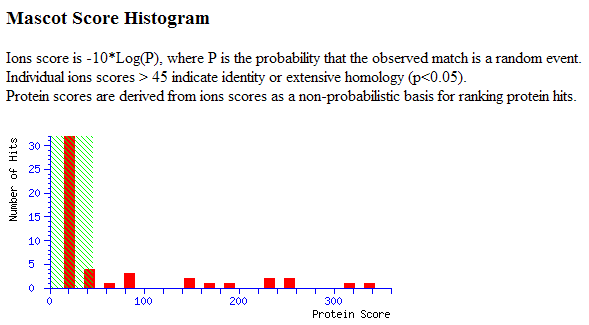


Matched peptide sequences: shown in Bold Red


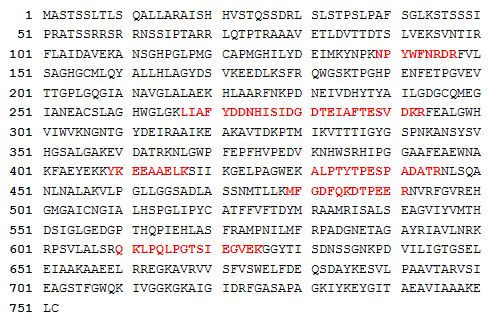


Matched peptides information:


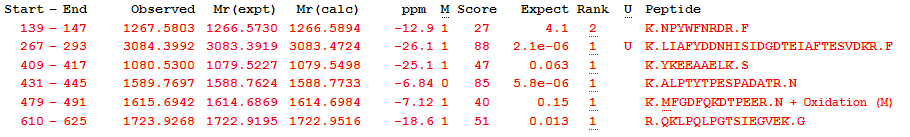


Spot No.: **7**

NCBI accession No.: gi | **147819511** Species: *Vitis vinifera*

PFF score: [256]

Protein name: hypothetical protein VITISV_037064

Matched peptides No.: [5] Sequence coverage %: [10]

Calculated Mr: **61387** Calculated *p*I: **5.20**

Probability Based Mow


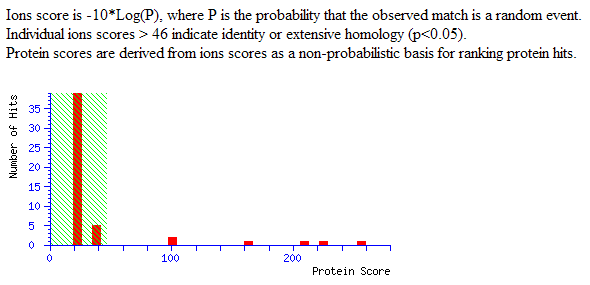


Matched peptide sequences: shown in Bold Red


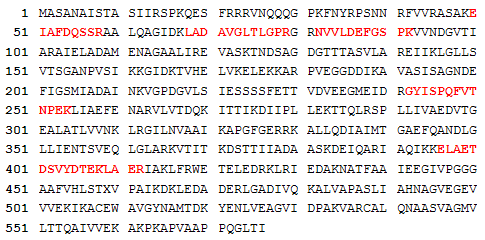


Matched peptides information:


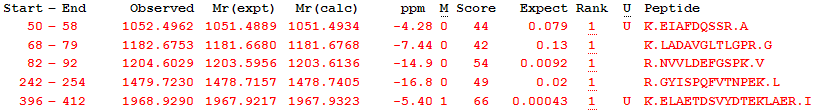


Spot No.: **8**

NCBI accession No.: gi | **3560664** Species: *Cymbidium ensifolium*

PFF score: [211]

Protein name: ribulose-1,5-bisphosphate carboxylase/oxygenase

Matched peptides No.: [5] Sequence coverage %: [13]

Calculated Mr: **49745** Calculated *p*I: **6.40**

Probability Based Mow


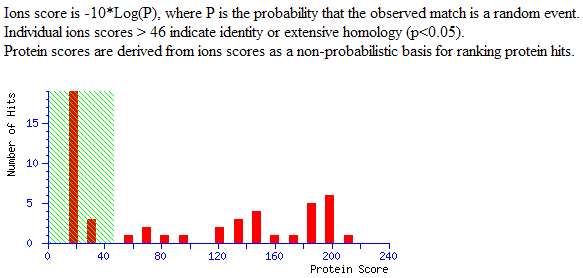


Matched peptide sequences: shown in Bold Red


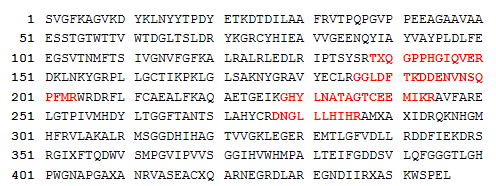


Matched peptides information:


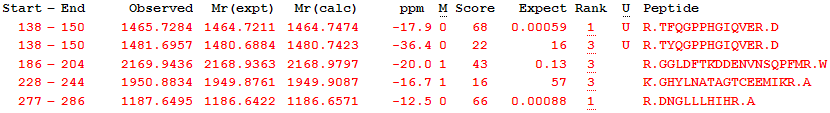


Spot No.: **9**

NCBI accession No.: gi | **225433375** Species: *Vitis vinifera*

PFF score: [204]

Protein name: chaperonin CPN60-2, mitochondrial isoform 1

Matched peptides No.: [6] Sequence coverage %: [14]

Calculated Mr: **61673** Calculated *p*I: **5.85**

Probability Based Mow


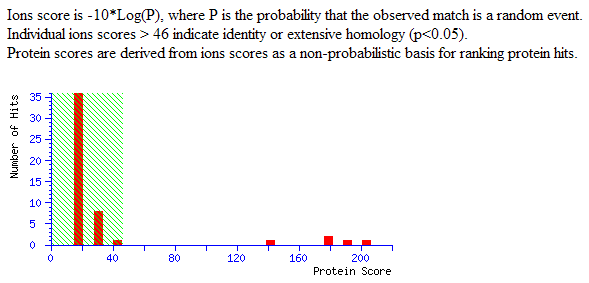


Matched peptide sequences: shown in Bold Red


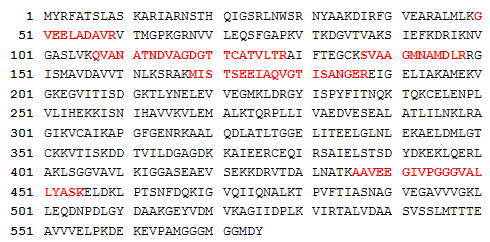


Matched peptides information:


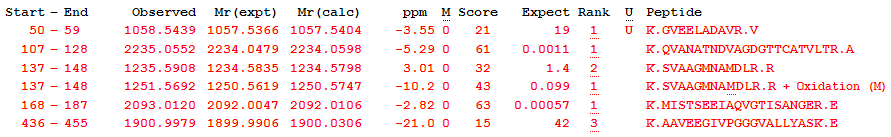


Spot No.: **10**

NCBI accession No.: gi | **40850676** Species: *Gossypium hirsutum*

PFF score: [124]

Protein name: betaine-aldehyde dehydrogenase

Matched peptides No.: [5] Sequence coverage %: [12]

Calculated Mr: **55372** Calculated *p*I: **5.60**

Probability Based Mow


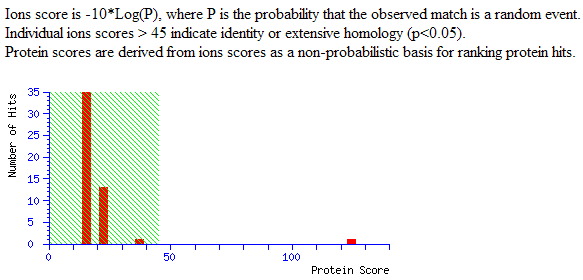


Matched peptide sequences: shown in Bold Red


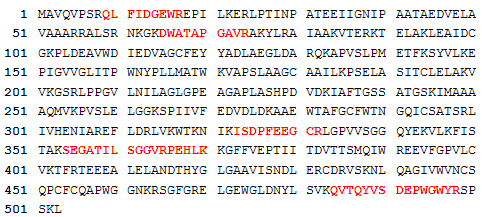


Matched peptides information:


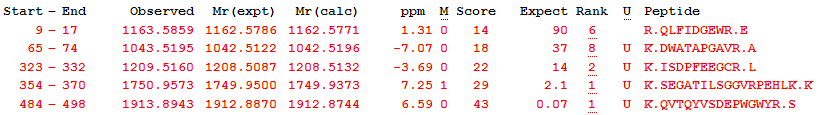


Spot No.: **11**

NCBI accession No.: gi | **91208909** Species: *Gossypium hirsutum*

PFF score: [939]

Protein name: ATP synthase CF1 beta subunit

Matched peptides No.: [13] Sequence coverage %: [42]

Calculated Mr: **53611** Calculated *p*I: **5.22**

Probability Based Mow


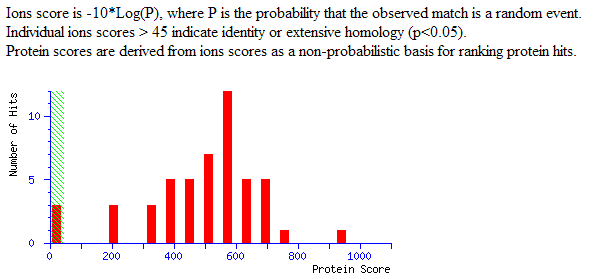


Matched peptide sequences: shown in Bold Red


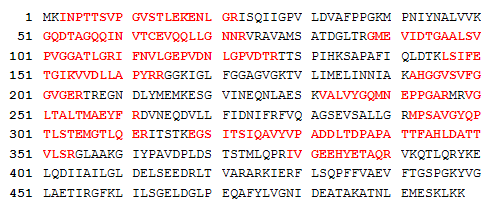


Matched peptides information:


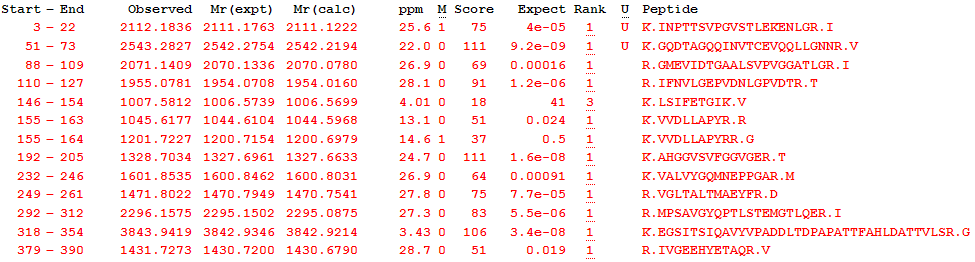


Spot No.: **12**

NCBI accession No.: gi | **255558986** Species: *Ricinus communis*

PFF score: [69]

Protein name: conserved hypothetical protein

Matched peptides No.: [2] Sequence coverage %: [2]

Calculated Mr: **50196** Calculated *p*I: **5.14**

Probability Based Mow


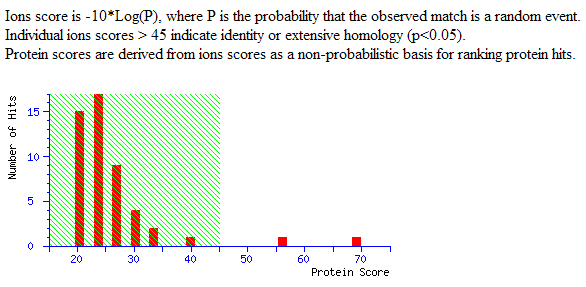


Matched peptide sequences: shown in Bold Red


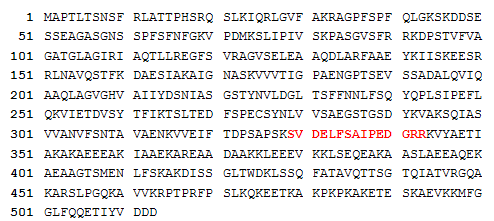


Matched peptides information:


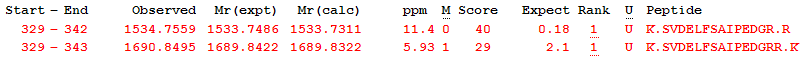


Spot No.: **13**

NCBI accession No.: gi | **255558986** Species: *Ricinus communis*

PFF score: [53]

Protein name: conserved hypothetical protein

Matched peptides No.: [2] Sequence coverage %: [2]

Calculated Mr: **54872** Calculated *p*I: **8.23**

Probability Based Mow


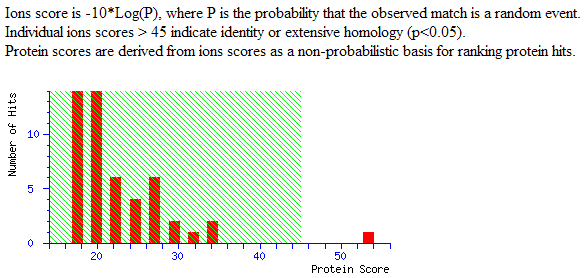


Matched peptide sequences: shown in Bold Red


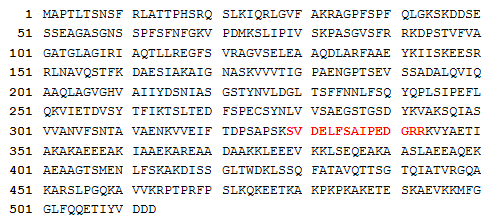


Matched peptides information:


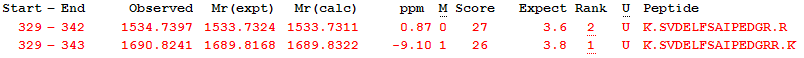


Spot No.: **14**

NCBI accession No.: gi | **11230404** Species: *Coffea sp. Pettersson*

PFF score: [998]

Protein name: ribulose-1,5-bisphosphate carboxylase/oxygenase large subunit

Matched peptides No.: [12] Sequence coverage %: [40]

Calculated Mr: **52917** Calculated *p*I: **5.96**

Probability Based Mow


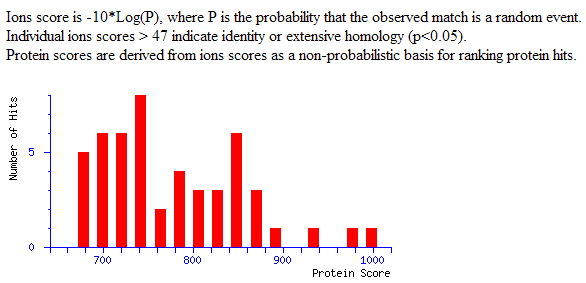


Matched peptide sequences: shown in Bold Red


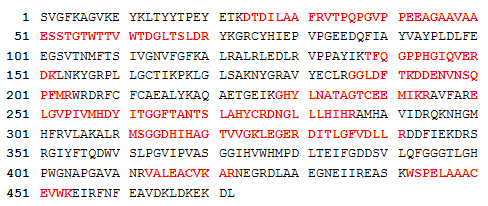


Matched peptides information:


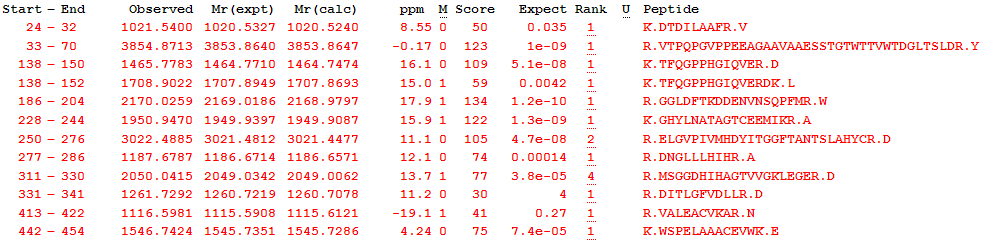


Spot No.: **15**

NCBI accession No.: gi | **33415263** Species: *Gossypium hirsutum*

PFF score: [502]

Protein name: enolase

Matched peptides No.: [11] Sequence coverage %: [36]

Calculated Mr: **47873** Calculated *p*I: **6.16**

Probability Based Mow


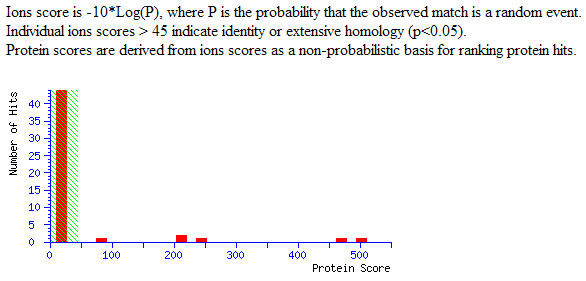


Matched peptide sequences: shown in Bold Red


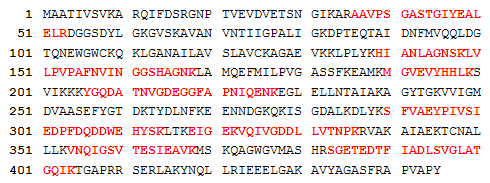


Matched peptides information:


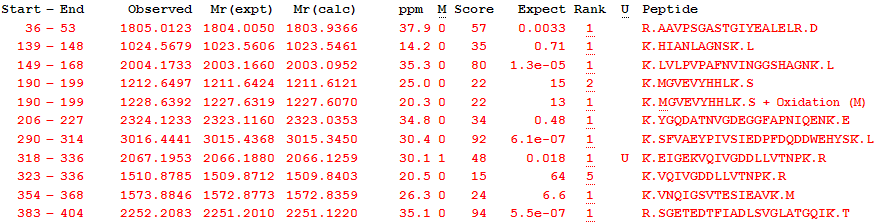


Spot No.: **16**

NCBI accession No.: gi | **12620883** Species: *Gossypium hirsutum*

PFF score: [822]

Protein name: ribulose-1,5-bisphosphate carboxylase/oxygenase activase 2

Matched peptides No.: [13] Sequence coverage %: [41]

Calculated Mr: **48609** Calculated *p*I: **5.06**

Probability Based Mow


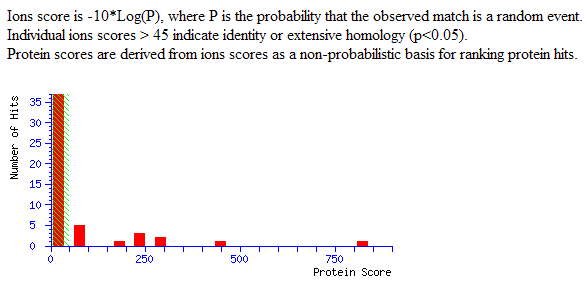


Matched peptide sequences: shown in Bold Red


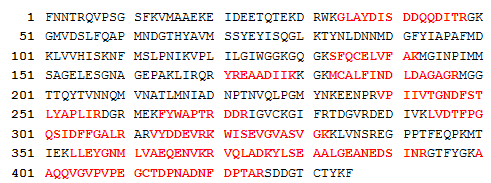


Matched peptides information:


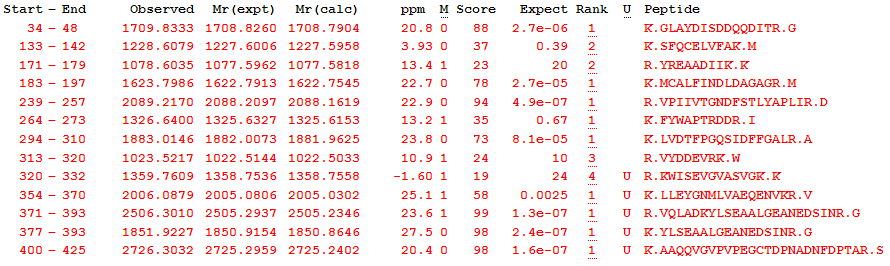


Spot No.: **17**

NCBI accession No.: gi | **281485191** Species: *Persea americana*

PFF score: [984]

Protein name: actin

Matched peptides No.: [12] Sequence coverage %: [46]

Calculated Mr: **41854** Calculated *p*I: **5.31**

Probability Based Mow


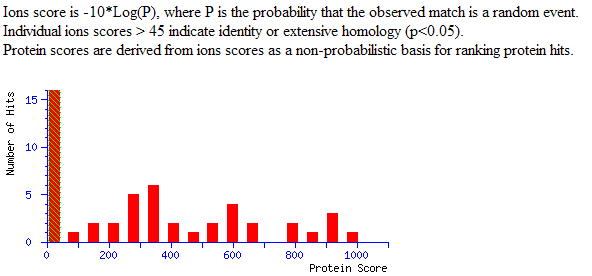


Matched peptide sequences: shown in Bold Red


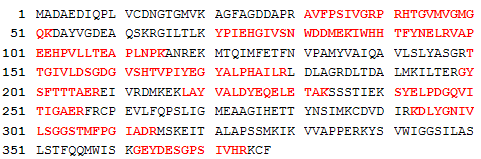


Matched peptides information:


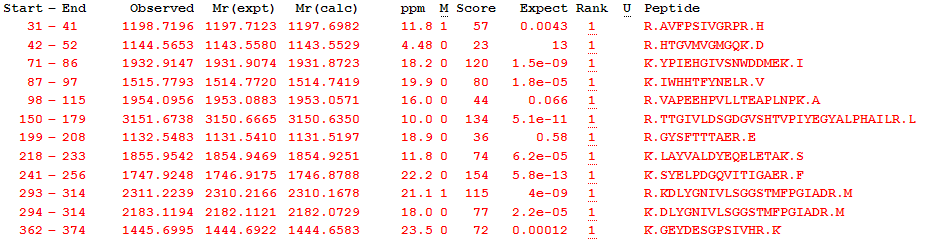


Spot No.: **18**

NCBI accession No.: gi | **211906462** Species: *Gossypium hirsutum*

PFF score: [160]

Protein name: glutamine synthase

Matched peptides No.: [5] Sequence coverage %: [16]

Calculated Mr: **39360** Calculated *p*I: **5.77**

Probability Based Mow


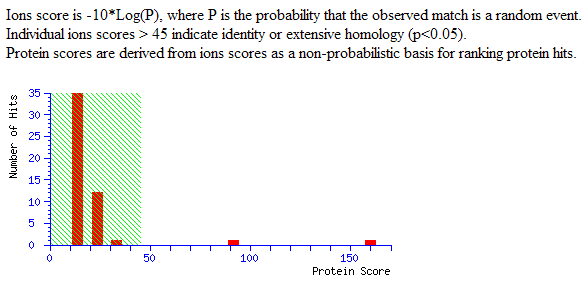


Matched peptide sequences: shown in Bold Red


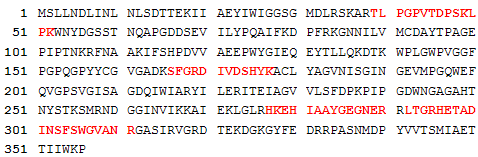


Matched peptides information:


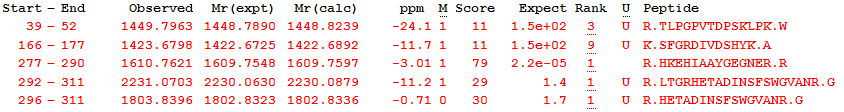


Spot No.: **19**

NCBI accession No.: gi | **449433772** Species: *Cucumis sativus*

PFF score: [150]

Protein name: magnesium-chelatase subunit ChlI, chloroplastic-like

Matched peptides No.: [5] Sequence coverage %: [15]

Calculated Mr: **46013** Calculated *p*I: **5.72**

Probability Based Mow


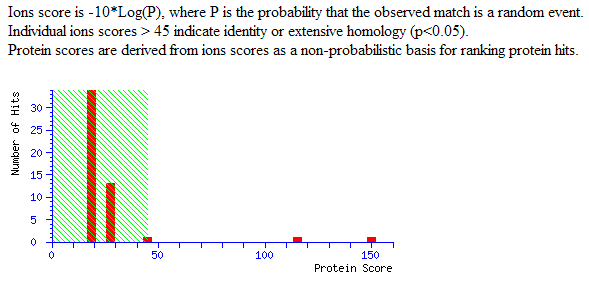


Matched peptide sequences: shown in Bold Red


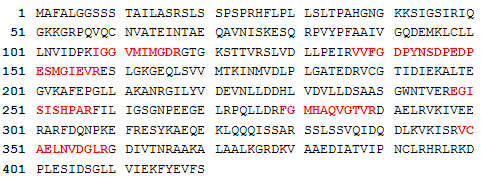


Matched peptides information:


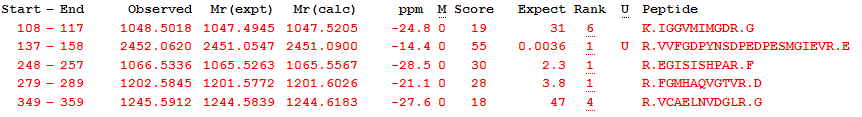


Spot No.20

NCBI accession No.: gi | **225451308** Species: *Vitis vinifera*

PFF score: [55]

Protein name: auxin-induced protein PCNT115

Matched peptides No.: [1] Sequence coverage %: [3]

Calculated Mr: **38039** Calculated *p*I: **5.65**

Probability Based Mow


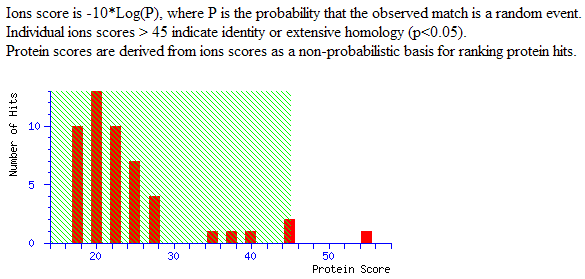


Matched peptide sequences: shown in Bold Red


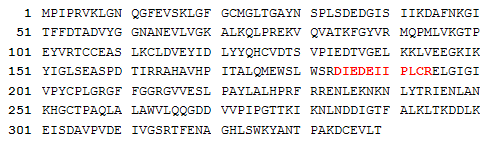


Matched peptides information:


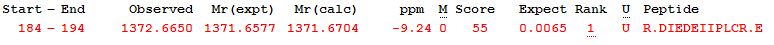


Spot No.: **21**

NCBI accession No.: gi | **255558669** Species: *Ricinus communis*

PFF score: [50]

Protein name: porphobilinogen deaminase, putative

Matched peptides No.: [3] Sequence coverage %: [10]

Calculated Mr: **40301** Calculated *p*I: **6.55**

Probability Based Mow


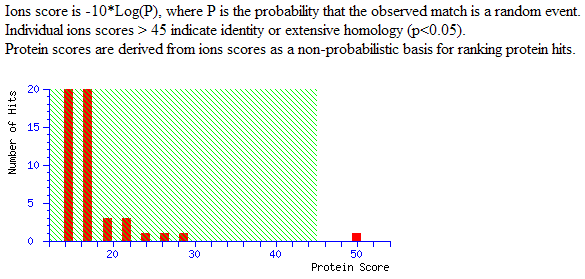


Matched peptide sequences: shown in Bold Red


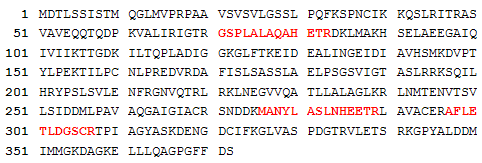


Matched peptides information:


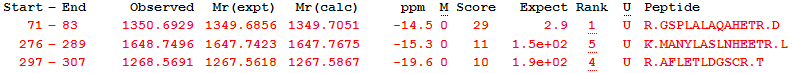


Spot No.: **22**

NCBI accession No.:gi|**329317332** Species:*Gossypium barbadense*

PFF score: [326]

Protein name: ribulose-1,5-bisphosphate carboxylase/oxygenase large subunit

Matched peptides No.: [5] Sequence coverage %: [16]

Calculated Mr: **53717** Calculated *p*I: **6.00**

Probability Based Mow


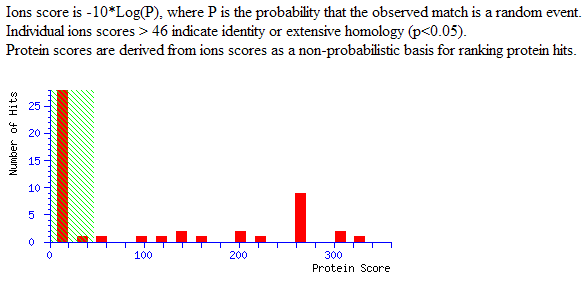


Matched peptide sequences: shown in Bold Red


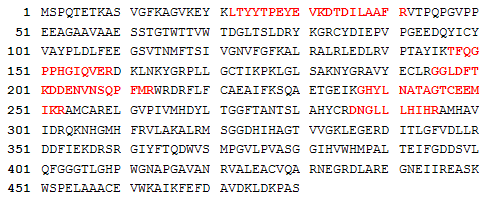


Matched peptides information:


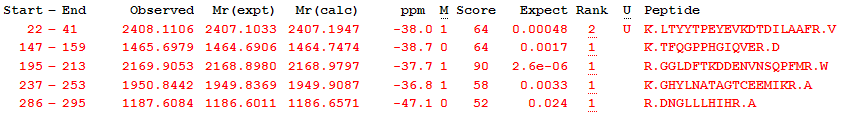


Spot No.:**23**

NCBI accession No.: gi | **225431122** Species: *Vitis vinifera*

PFF score: [270]

Protein name: ferredoxin--NADP reductase, leaf isozyme, chloroplastic isoform 1

Matched peptides No.: [7] Sequence coverage %: [22]

Calculated Mr: **40793** Calculated *p*I: **8.91**

Probability Based Mow


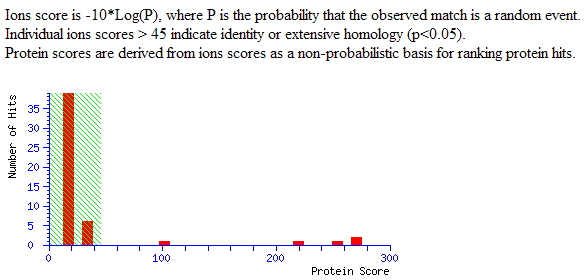


Matched peptide sequences: shown in Bold Red


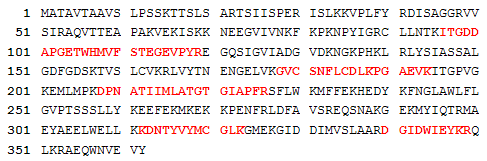


Matched peptides information:


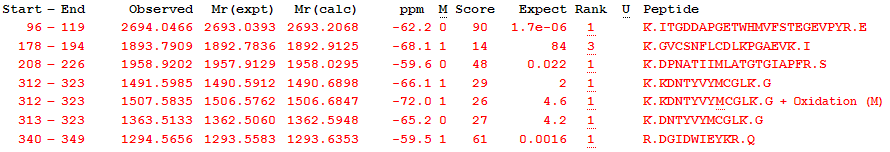


Spot No.: **24**

NCBI accession No.: gi | **449442663** Species: *Cucumis sativus*

PFF score: [442]

Protein name: phosphoglycolate phosphatase-like

Matched peptides No.: [8] Sequence coverage %: [26]

Calculated Mr: **41716** Calculated *p*I: **6.47**

Probability Based Mow


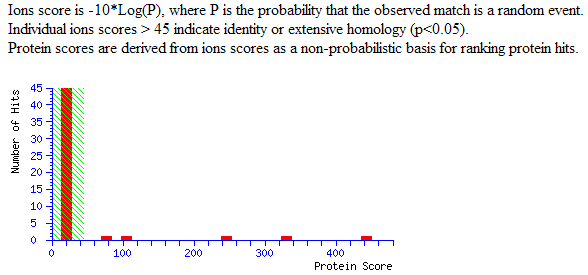


Matched peptide sequences: shown in Bold Red


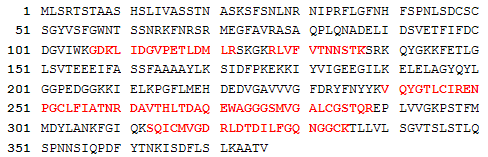


Matched peptides information:


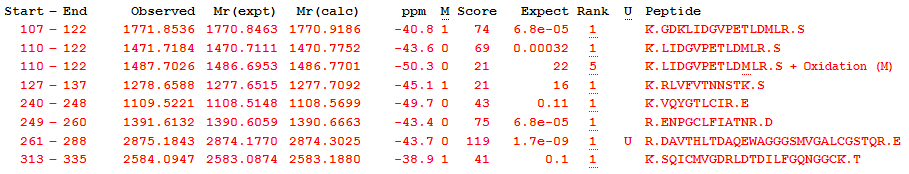


Spot No.: **25**

NCBI accession No.: gi|**292668595** Species:*Eschscholzia californica*

PFF score: [104]

Protein name: sanguinarine reductase

Matched peptides No.: [2] Sequence coverage %: [6]

Calculated Mr: **29615** Calculated *p*I: **4.97**

Probability Based Mow


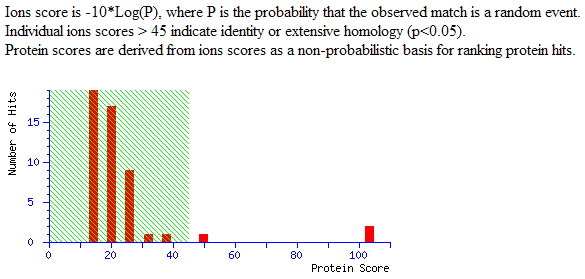


Matched peptide sequences: shown in Bold Red


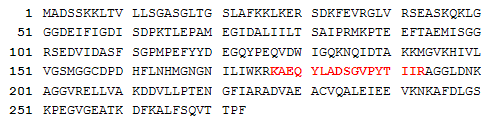


Matched peptides information:


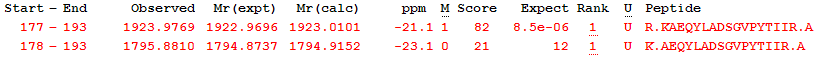


Spot No.: **26**

NCBI accession No.: gi | **188149** Species: *Plocama pendula*

PFF score: [366]

Protein name: ribulose-1,5-bisphosphate carboxylase/oxygenase large subunit

Matched peptides No.: [9] Sequence coverage %: [22]

Calculated Mr: **52615** Calculated *p*I: **6.61**

Probability Based Mow


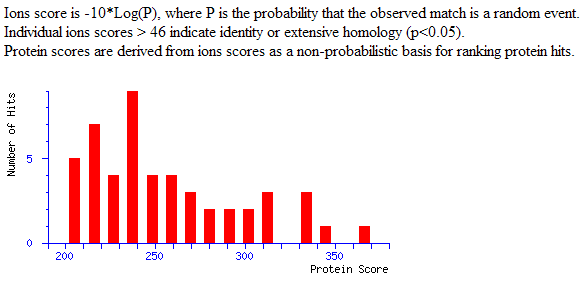


Matched peptide sequences: shown in Bold Red


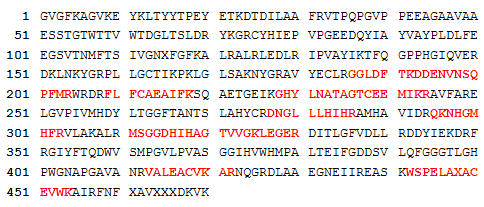


Matched peptides information:


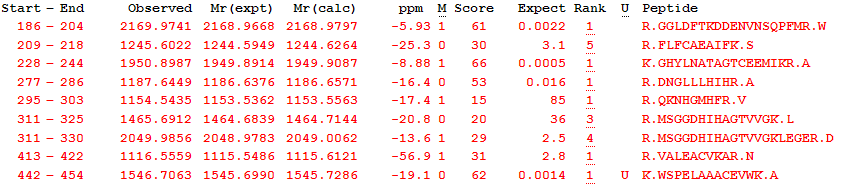


Spot No.: **27**

NCBI accession No.: gi | **14594915** Species: *Nicotiana tabacum*

PFF score: [113]

Protein name: putative alpha3 proteasome subunit

Matched peptides No.: [3] Sequence coverage %: [32]

Calculated Mr: **18240** Calculated *p*I: **6.12**

Probability Based Mow


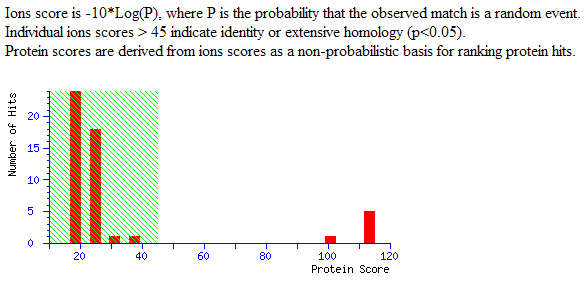


Matched peptide sequences: shown in Bold Red


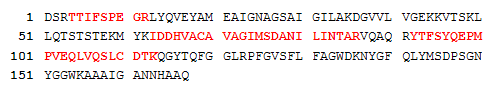


Matched peptides information:


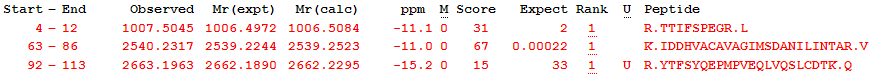


Spot No.:**28**

NCBI accession No.: gi | **226358407** Species: *Gossypium hirsutum*

PFF score: [166]

Protein name: chloroplast chlorophyll A-B binding protein

Matched peptides No.: [3] Sequence coverage %: [20]

Calculated Mr: **25581** Calculated *p*I: **5.53**

Probability Based Mow


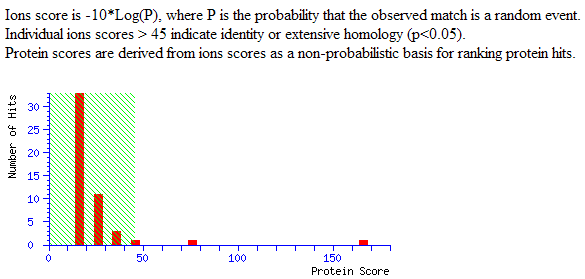


Matched peptide sequences: shown in Bold Red


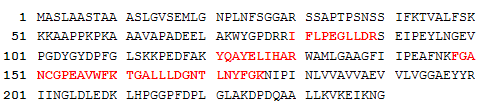


Matched peptides information:


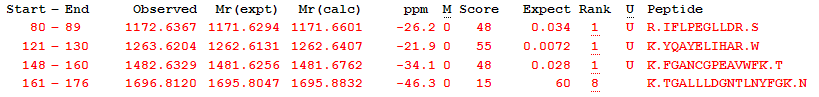


Spot No.: **29**

NCBI accession No.: gi | **118489712** Species: *Populus trichocarpa*

PFF score: [156]

Protein name: unknown

Matched peptides No.: [3] Sequence coverage %: [9]

Calculated Mr: **24420** Calculated *p*I: **4.77**

Probability Based Mow


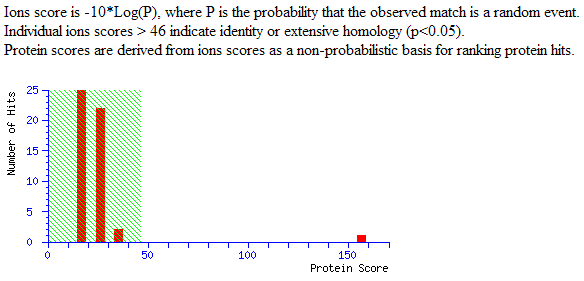


Matched peptide sequences: shown in Bold Red


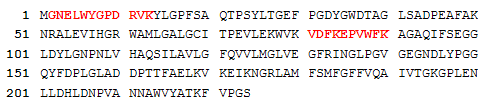


Matched peptides information:


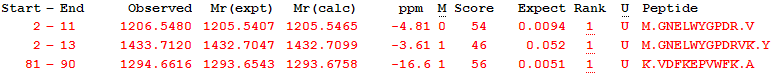


Spot No.: **30**

NCBI accession No.: gi | **147767601** Species: *Vitis vinifera*

PFF score: [59]

Protein name: hypothetical protein VITISV_003162

Matched peptides No.: [2] Sequence coverage %: [11]

Calculated Mr: **25523** Calculated *p*I: **8.46**

Probability Based Mow

Matched peptide sequences: shown in Bold Red


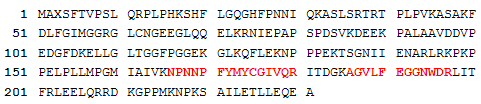


Matched peptides information:


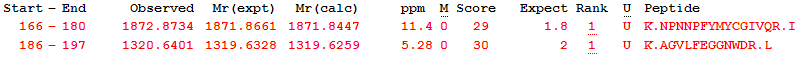


Spot No.: **31**

NCBI accession No.: gi | **302595736** Species: *（-）*

PFF score: [215]

Protein name: Oxygen-evolving enhancer protein 2

Matched peptides No.: [3] Sequence coverage %: [9]

Calculated Mr: **28231** Calculated *p*I: **8.67**

Probability Based Mow


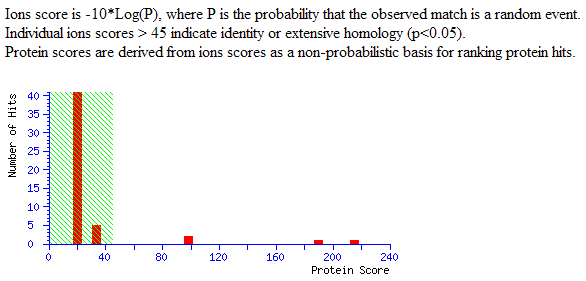


Matched peptide sequences: shown in Bold Red


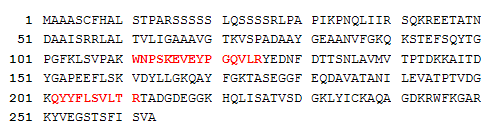


Matched peptides information:


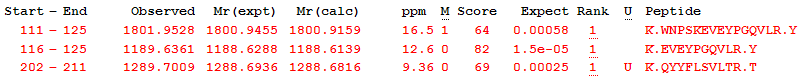


Spot No.: **32**

NCBI accession No.: gi | **548699** Species: *（-）*

PFF score: [104]

Protein name: Ribulose bisphosphate carboxylase large chain

Matched peptides No.: [4] Sequence coverage %: [9]

Calculated Mr: **52637** Calculated *p*I: **6.12**

Probability Based Mow


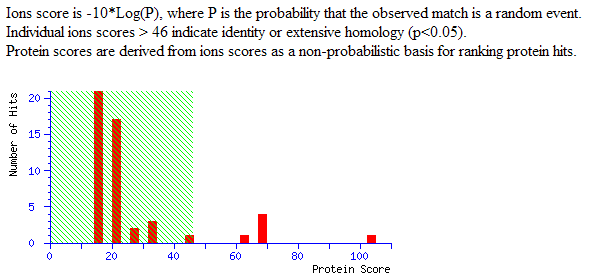


Matched peptide sequences: shown in Bold Red


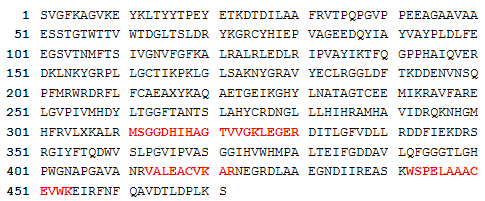


Matched peptides information:


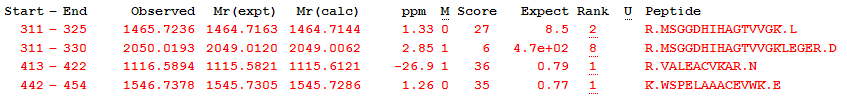


Spot No.: **33**

NCBI accession No.: gi | **315364830** Species: *Citrullus lanatus*

PFF score: [231]

Protein name: chloroplast Rieske-type ion-sulfur protein

Matched peptides No.: [3] Sequence coverage %: [18]

Calculated Mr: **24602** Calculated *p*I: **8.45**

Probability Based Mow


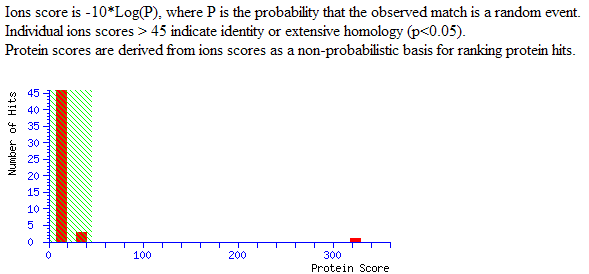


Matched peptide sequences: shown in Bold Red


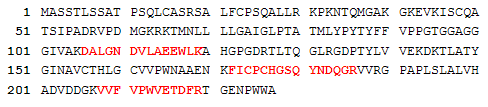


Matched peptides information:


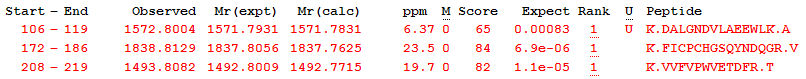


Spot No.: **34**

NCBI accession No.: gi | **225457446** Species: *Vitis vinifera*

PFF score: [147]

Protein name: nucleoside diphosphate kinase 2, chloroplastic isoform 1

Matched peptides No.: [3] Sequence coverage %: [13]

Calculated Mr: **25971** Calculated *p*I: **9.28**

Probability Based Mow


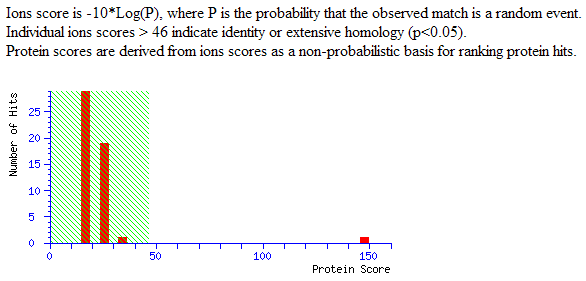


Matched peptide sequences: shown in Bold Red


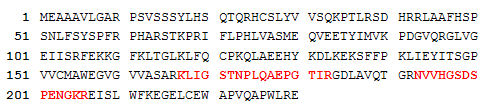


Matched peptides information:

Spot No.: **35**

NCBI accession No.: gi | **211906510** Species: *Gossypium hirsutum*

PFF score: [116]

Protein name: major latex-like protein

Matched peptides No.: [3] Sequence coverage %: [27]

Calculated Mr: **17163** Calculated *p*I: **5.46**

Probability Based Mow

Matched peptide sequences: shown in Bold Red

Matched peptides information:
