# Supplementary material for: Comparative proteomics of Bt-transgenic and non-transgenic cotton leaves
Source: Proteome Sci. 2015 May 2;13:15. doi: 10.1186/s12953-015-0071-8 (PMC4422549; doi:10.1186/s12953-015-0071-8)

**Additional File 5:**

**the main pathways involved in transgenic cotton**

**Sequence distribution: biological _ process**


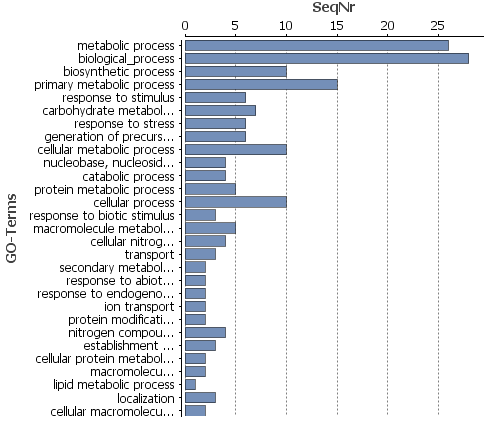


**GO**-**level distribution**


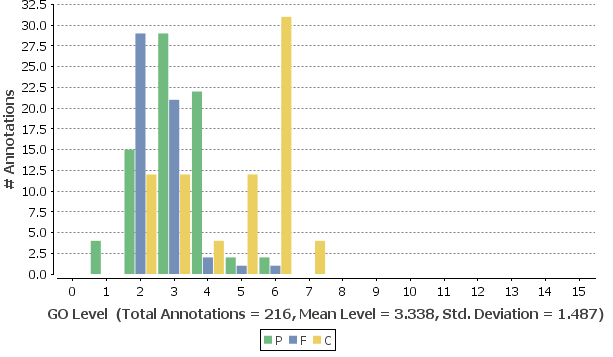


**Number of GO-terms for sequences with length(x)**


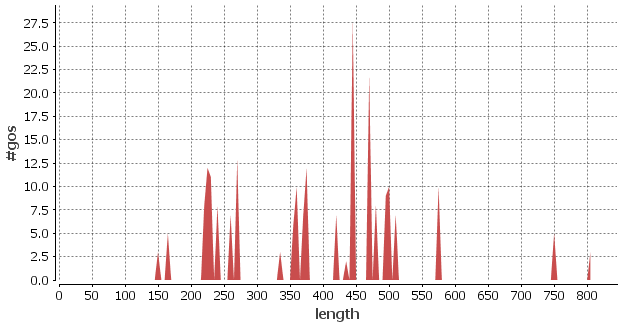


**Annotation-Score distribution**

**
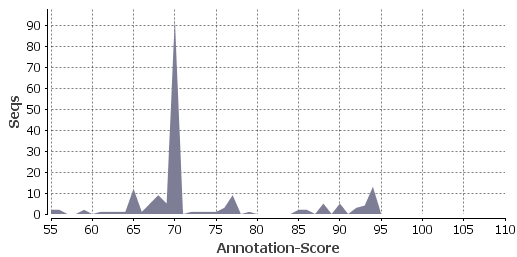
**

**The main pathways**

Carbon fixation in photosynthetic organisms: 7 proteins


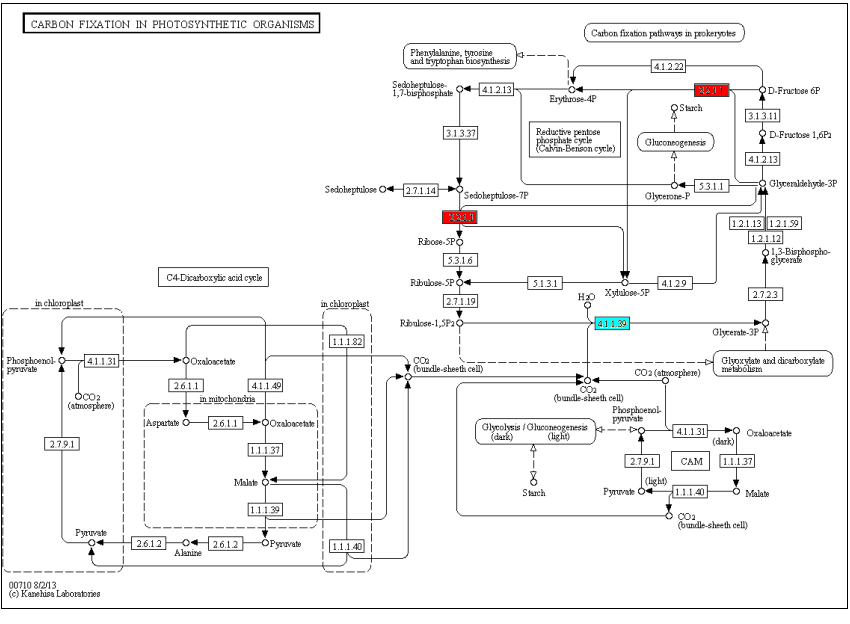


Glyoxylate and dicarboxylate metabolism: 7 proteins


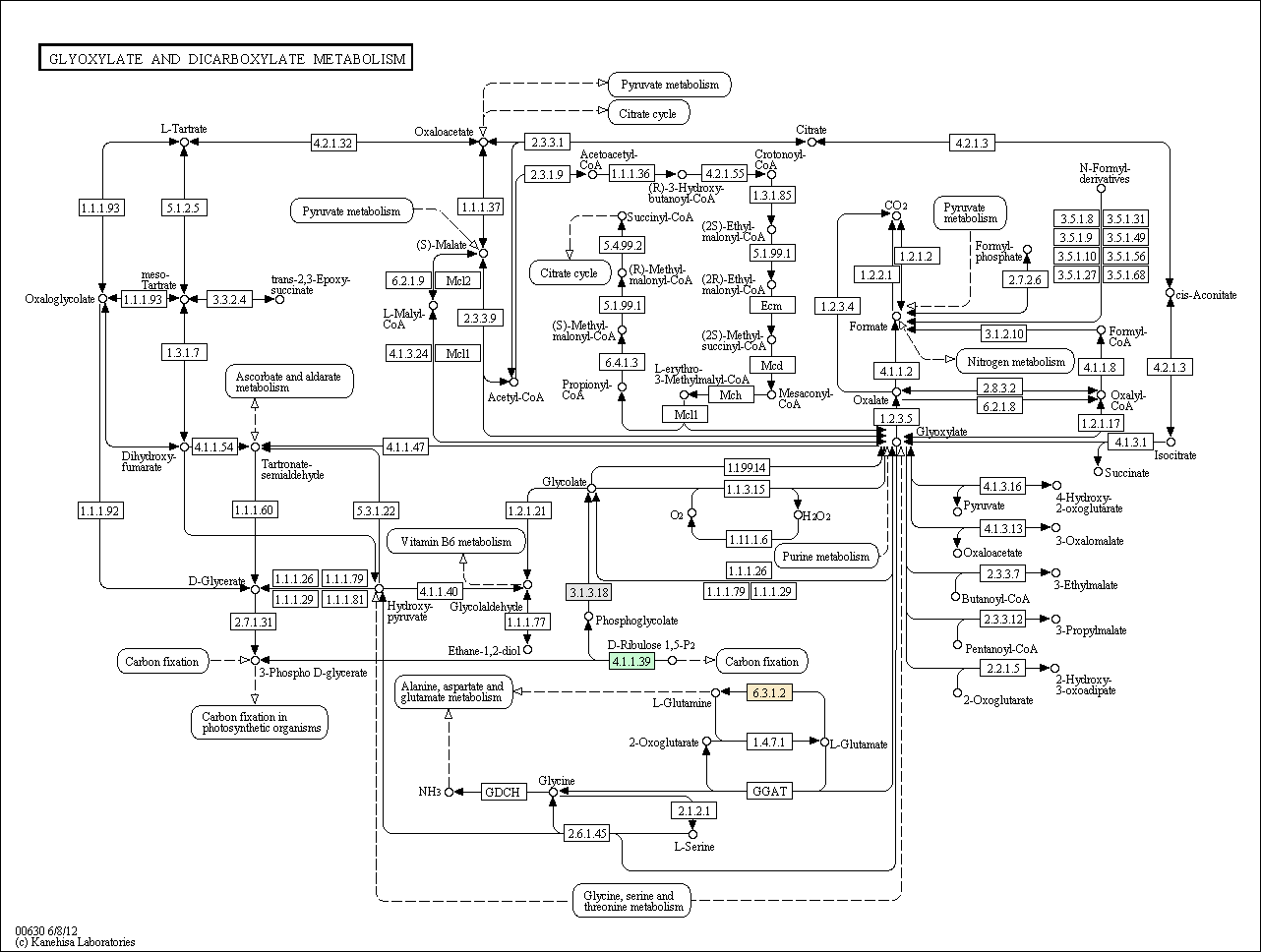


Purine metabolism: 2 proteins


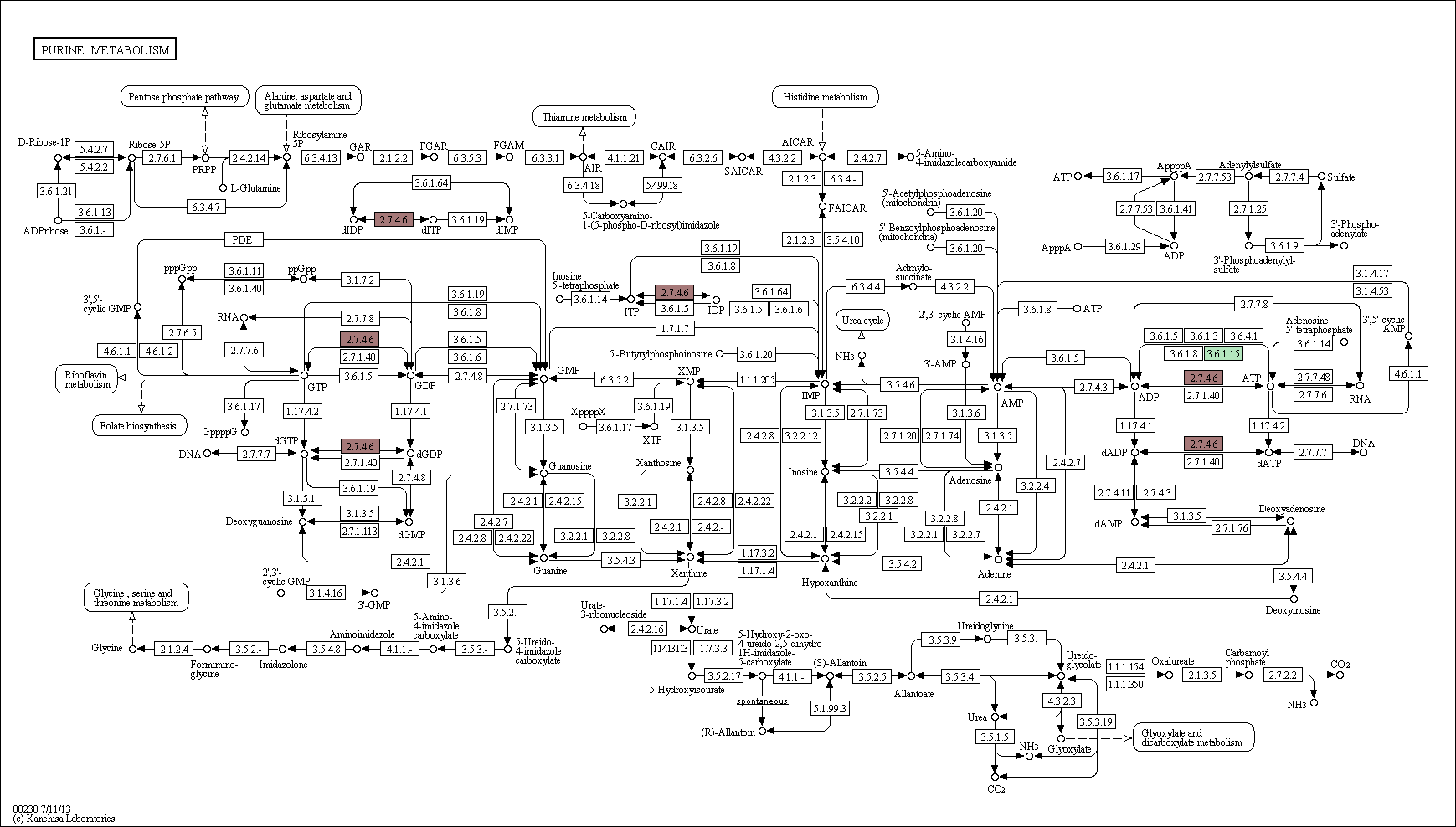


Pentose phosphate pathway: 2 proteins


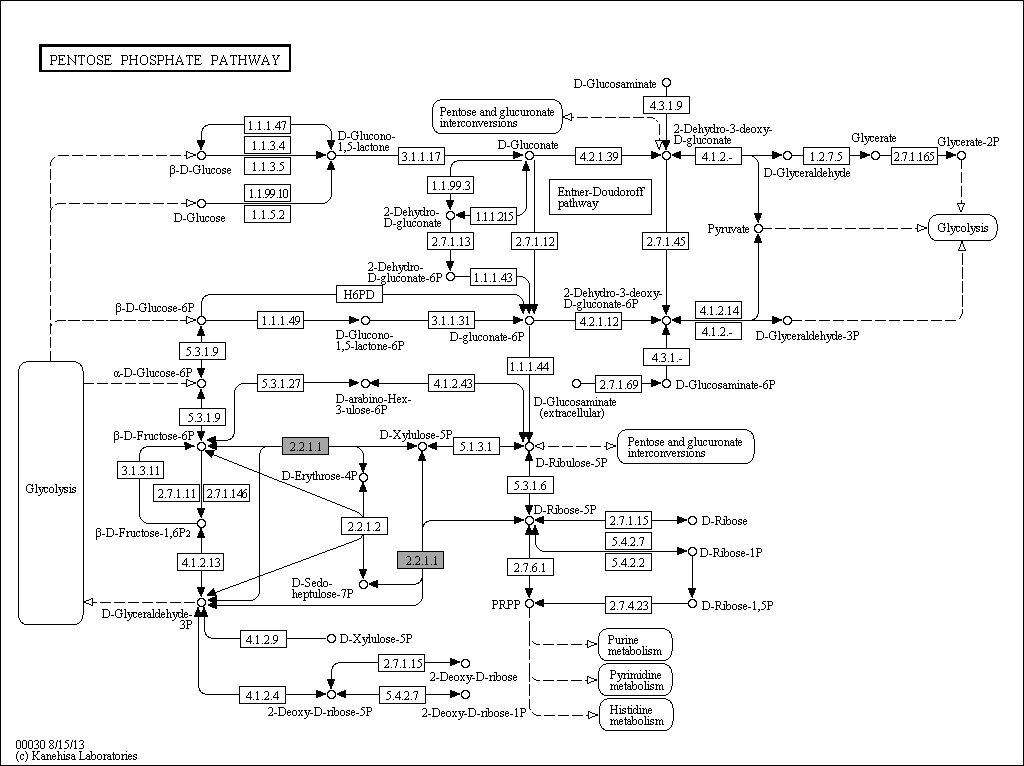


Nitrogen metabolism: 2 proteins


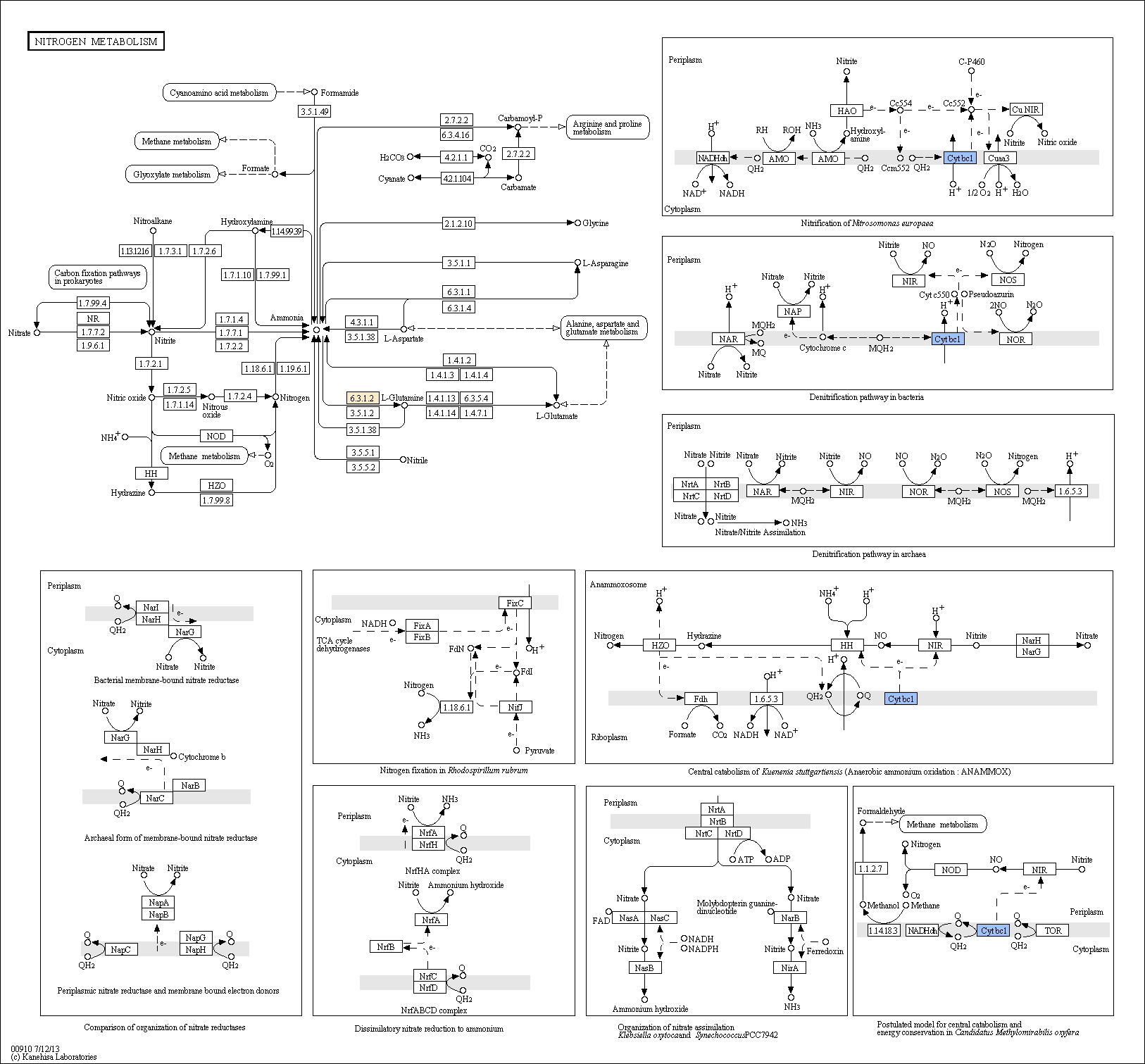


Photosynthesis: 2 proteins


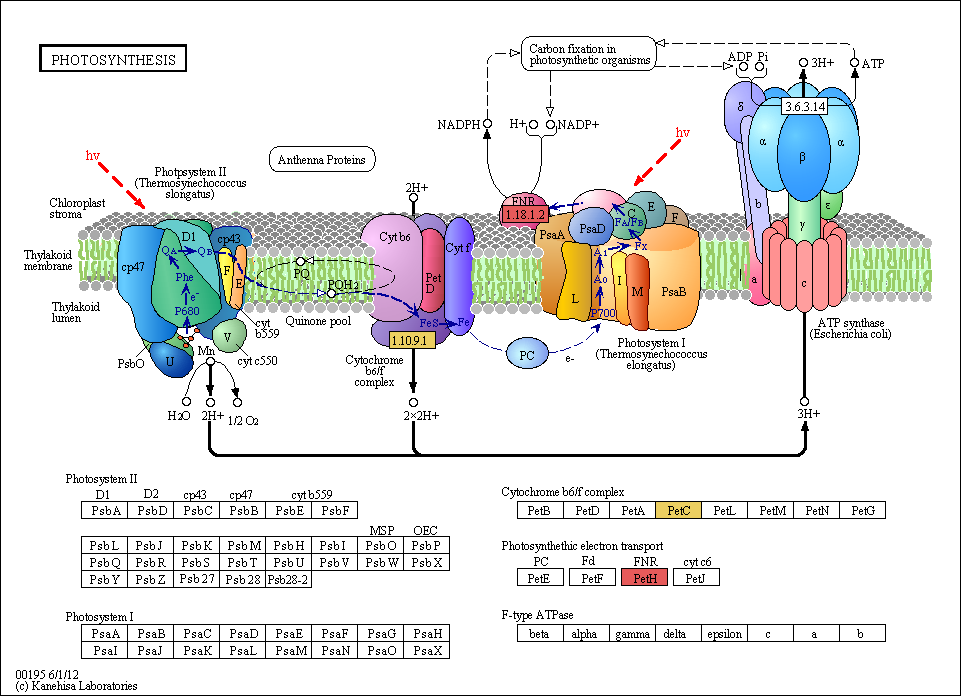


Porphyrin and chlorophyll metabolism: 2 proteins


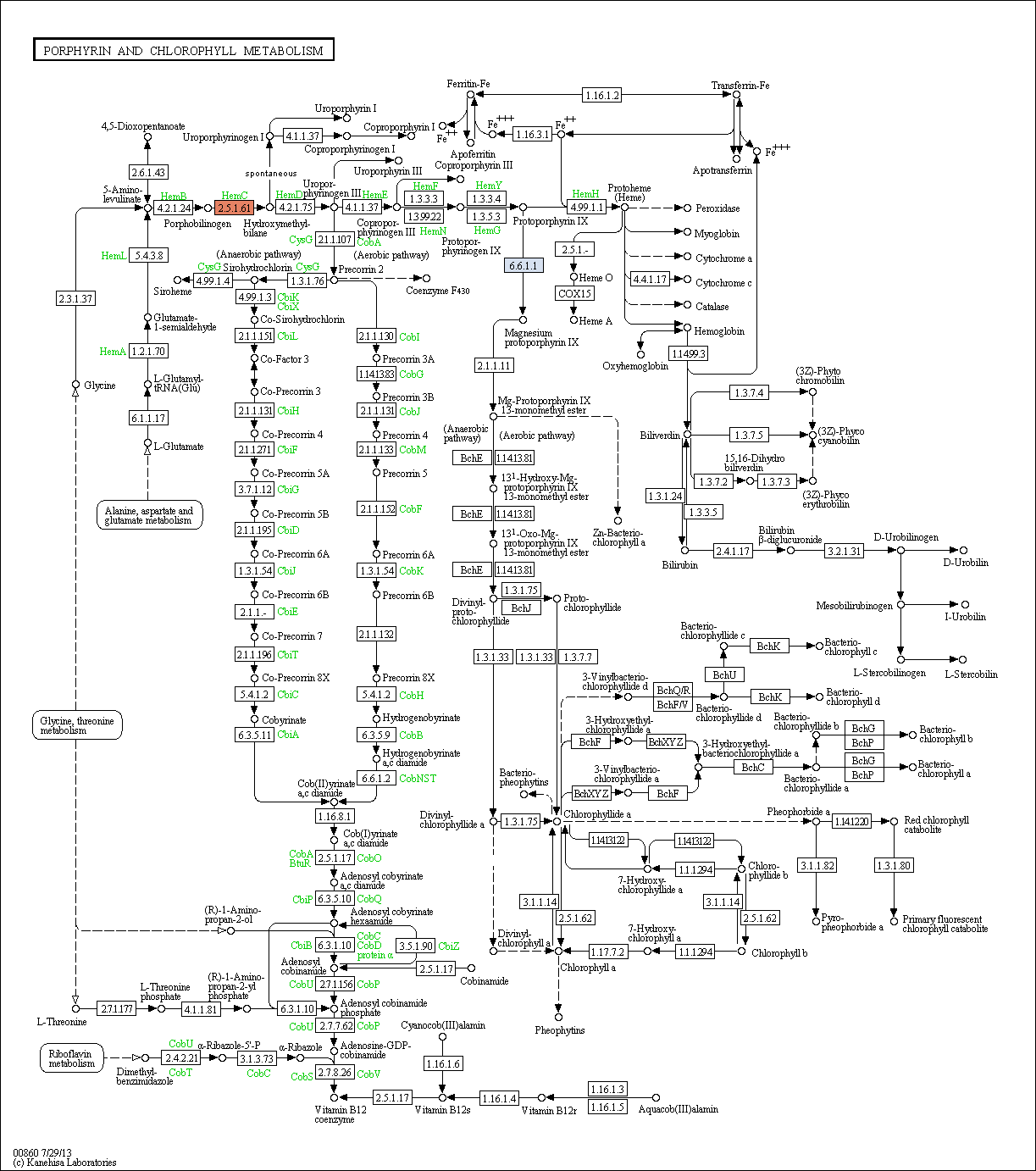


Oxidative phosphorylation: 2 proteins


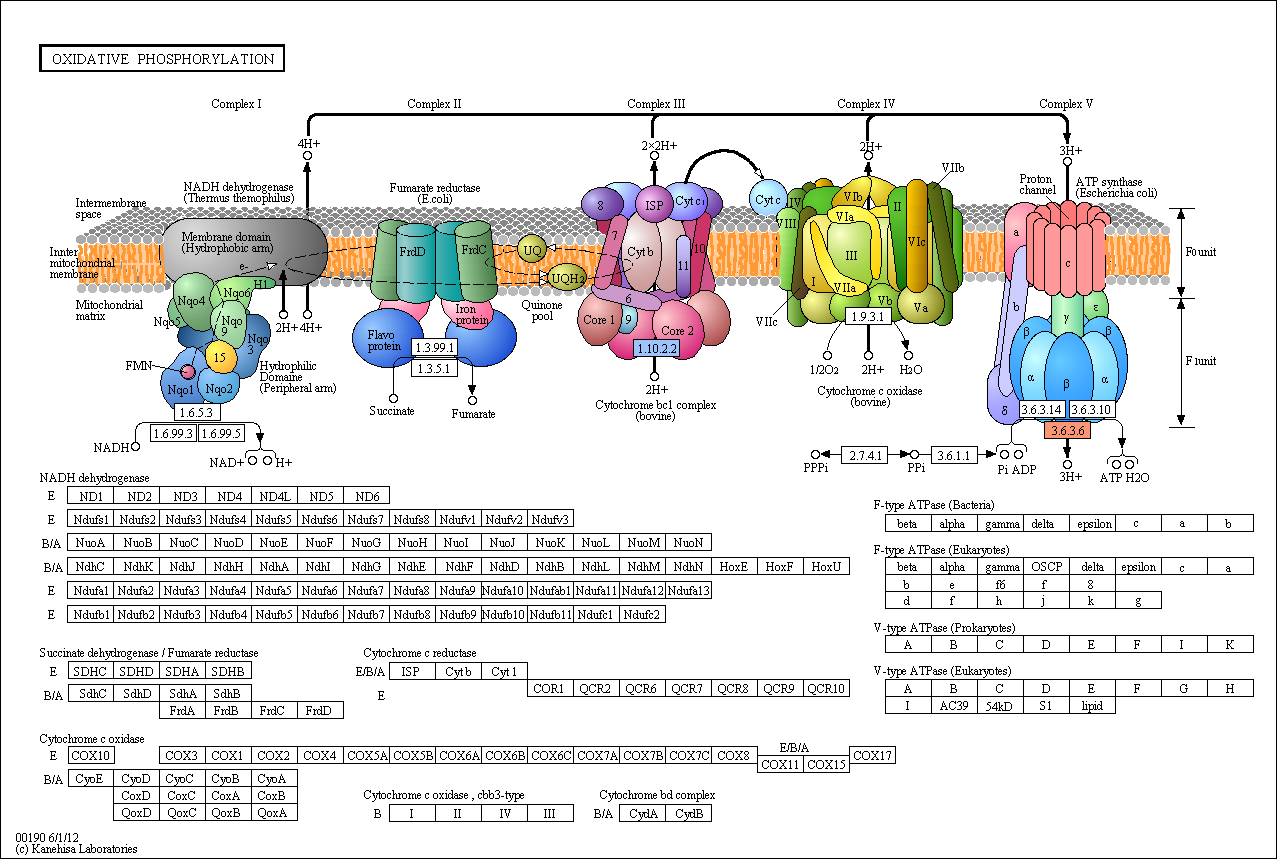


Biosynthesis of ansamycins: 2 proteins


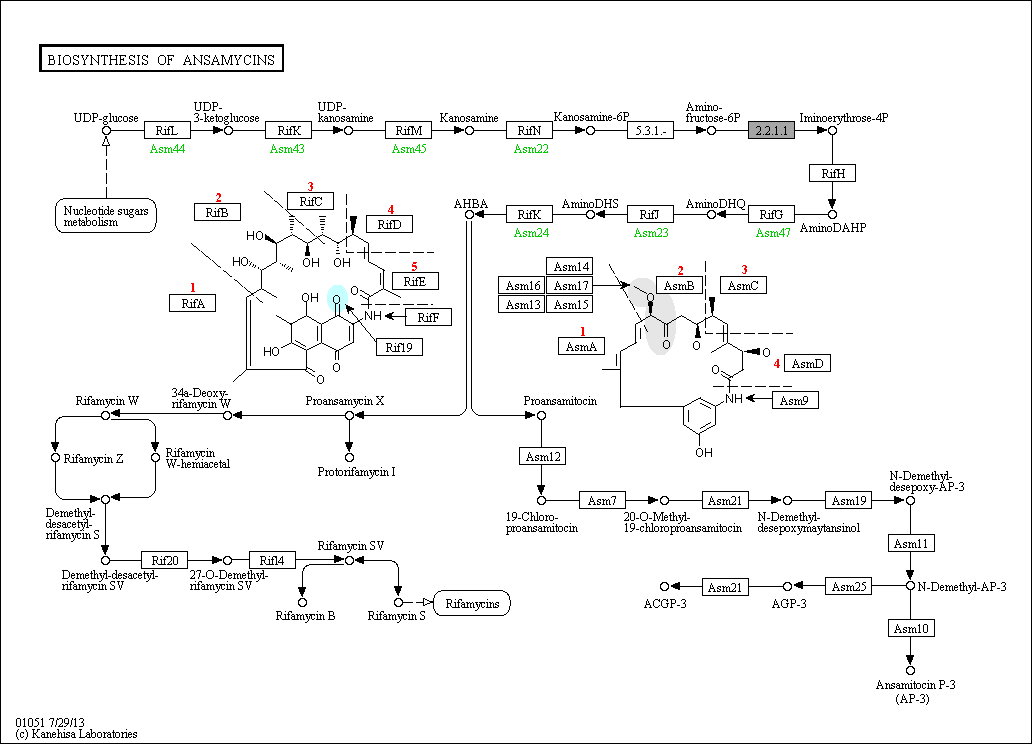

Supplement: Additional file 5: — The main pathways involved in transgenic cotton. [file 12953_2015_71_MOESM5_ESM.doc]
