# Supplementary material for: Comparative proteomics of Bt-transgenic and non-transgenic cotton leaves
Source: Proteome Sci. 2015 May 2;13:15. doi: 10.1186/s12953-015-0071-8 (PMC4422549; doi:10.1186/s12953-015-0071-8)
Supplement: Additional file 6: — Primers used in qRT-PCR. [file 12953_2015_71_MOESM6_ESM.doc]

**Additional File 6: Primers used in qRT-PCR**

**Spot 1**

| C1F: 5’-CGAGGTCGGTTATGATGATGTT-3’ |
| --- |
| C1R: 5’- CCAGGAGGTCCGTAAAGCAG-3’ |

**Spot 7**

| C7F: 5’-CAACAAGGGGAGTTTAAGGAGG-3’ |
| --- |
| C7F: 5’-AGTGTAAGGCCAACAGCATCAG-3’ |

**Spot 8**

| C8F: 5’-GCATTTCCCCAAGGGTGTC-3’ |
| --- |
| C8F: 5’-GGGTTTCTATGCCAGGTGTTCT-3’ |

**Spot 9**

| C9F: 5’-AGTTGGACAAGCTACCTACGGC-3’ |
| --- |
| C9F: 5’-GCATCATAGCCAAGATCAGGGT-3’ |

**Spot 10**

| C10F: 5’-TGGCTTTGGGCGTGAACT-3’ |
| --- |
| C10F: 5’-ACAGCTTTGATGGTGAGCGATA-3’ |

**Spot 11**

| C11F: 5’-TCAGGAACGTATTACCACCACC -3’ |
| --- |
| C11F: 5’-GTCAAGTCATCAGCAGGCACA-3’ |

**Spot 14**

| C14F: 5’-CACGTCCCTCATTACGAGCTT-3’ |
| --- |
| C14F: 5’-GGGTTTCTATGCCAGGTGTTCT-3’ |

**Spot 15**

| C15F: 5’-AGGTCTGAGCGTTTAGCCAAGT-3’ |
| --- |
| C15F: 5’-CGCATCCAGTCTCAGCACATT-3’ |

**Spot 16**

| C16F: 5’-CTGGGTATTTGGGGAGGCA -3’ |
| --- |
| C16F: 5’-GTTGACGGTGTATTGGGTGGT-3’ |

**Spot 18**

| C18F: 5’-TACATCAATGATTGCCGAGACC-3’ |
| --- |
| C18F: 5’-CCACTGGCCCAACAAAGAAA-3’ |

**Spot 19**

| C19F: 5’-TGCTCGGTTTATTCTCATCGG-3’ |
| --- |
| C19F: 5’-GCTCTGCCTTGTAAGAATCACG-3’ |

**Spot 20**

| C1F: 5’-TCAATGCACTCCGGCTCAA-3’ |
| --- |
| C1F: 5’-CAGGAATCGGTACAACATCGTC-3’ |

**Spot 21**

| C21F: 5’-GCCTTTCTGTTGACACTTGACG-3’ |
| --- |
| C21F: 5’-TTCTTTGCCAGCATCCTTCC-3’ |

**Spot 22**

| C22F: 5’-AGGGAGGGACTTATGTCACCAC-3’ |
| --- |
| C22F: 5’-ACTCGGAAGGCTGCCAAGA-3’ |

**Spot 23**

| C23F: 5’-CTGAGCAATGGAATGTGGAAGT-3’ |
| --- |
| C23F: 5’-ATCAAAGCTGAAGCATGGGATA-3’ |

**Spot 24**

| C24F: 5’-GTTTGGACAAAACGGTGGCT-3’ |
| --- |
| C24F: 5’-AAGGGCTTCTTGATCTGATGGT-3’ |

**Spot 27**

| C27F: 5’-TGGTGTTGTCTTGGTTGGTGA-3’ |
| --- |
| C27F: 5’-ACAGGCATTGGCTCTTGGTAA-3’ |

**Spot 28**

| C28F: 5’-ATCGGCTCCAACACCATCC-3’ |
| --- |
| C28F: 5’-GAGCAACGGTCTTGAAAATGG-3’ |

**Spot 29**

| C29F: 5’-TGGTGTTGGTGAAGGCAATG-3’ |
| --- |
| C29F: 5’-CCCAGGCACAAACTTAGTAGCA-3’ |

**Spot 30**

| C30F: 5’-ACCCGAACAACCCCTTCTACA-3’ |
| --- |
| C30F: 5’-AACCCCTGCTTTCCCATCA-3’ |
